# Increased autophagic sequestration in adaptor protein-3 deficient dendritic cells limits inflammasome activity and impairs antibacterial immunity

**DOI:** 10.1371/journal.ppat.1006785

**Published:** 2017-12-18

**Authors:** Adriana R. Mantegazza, Meghan A. Wynosky-Dolfi, Cierra N. Casson, Ariel J. Lefkovith, Sunny Shin, Igor E. Brodsky, Michael S. Marks

**Affiliations:** 1 Department of Pathology and Laboratory Medicine, Children's Hospital of Philadelphia, Philadelphia, PA, United States of America; 2 Department of Pathology and Laboratory Medicine, Perelman School of Medicine, University of Pennsylvania, Philadelphia, PA, United States of America; 3 Department of Physiology, Perelman School of Medicine, University of Pennsylvania, Philadelphia, PA, United States of America; 4 Department of Pathobiology, School of Veterinary Medicine, University of Pennsylvania, Philadelphia, PA, United States of America; 5 Department of Microbiology, Perelman School of Medicine, University of Pennsylvania, Philadelphia, PA, United States of America; University of California Davis School of Medicine, UNITED STATES

## Abstract

Bacterial pathogens that compromise phagosomal membranes stimulate inflammasome assembly in the cytosol, but the molecular mechanisms by which membrane dynamics regulate inflammasome activity are poorly characterized. We show that in murine dendritic cells (DCs), the endosomal adaptor protein AP-3 –which optimizes toll-like receptor signaling from phagosomes–sustains inflammasome activation by particulate stimuli. AP-3 independently regulates inflammasome positioning and autophagy induction, together resulting in delayed inflammasome inactivation by autophagy in response to *Salmonella* Typhimurium (STm) and other particulate stimuli specifically in DCs. AP-3-deficient DCs, but not macrophages, hyposecrete IL-1β and IL-18 in response to particulate stimuli *in vitro*, but caspase-1 and IL-1β levels are restored by silencing autophagy. Concomitantly, AP-3-deficient mice exhibit higher mortality and produce less IL-1β, IL-18, and IL-17 than controls upon oral STm infection. Our data identify a novel link between phagocytosis, inflammasome activity and autophagy in DCs, potentially explaining impaired antibacterial immunity in AP-3-deficient patients.

## Author summary

Bacterial uptake by phagocytic cells such as dendritic cells (DCs) stimulates signaling from membrane-bound toll-like receptors (TLRs) to shape adaptive immune responses. Pathogenic bacteria that damage phagocytic membranes additionally stimulate the cytoplasmic inflammasome, producing the highly inflammatory cytokines IL-1β and IL-18. Host molecular mechanisms that link phagosomal signaling to inflammasome regulation are poorly characterized. We show that in DCs, the endosomal adaptor protein-3 (AP-3) complex optimizes phagocytosis-induced inflammasome activity by two mechanisms: AP-3 promotes TLR signaling-dependent transcription of inflammasome components and antagonizes autophagy-dependent inflammasome silencing. Consequently, AP-3 deficient DCs hyposecrete IL-1β and IL-18 in response to phagocytosed stimuli, and AP-3 deficient mice succumb to infection by a bacterial pathogen. AP-3 thus links phagosome signaling, inflammasome activity and autophagy in DCs.

## Introduction

Dendritic cells (DCs) sense and integrate innate immune signals to stimulate adaptive T cell immune responses. To elicit effective defense against pathogens and prevent abnormal responses to non-pathogenic microorganisms, DCs adjust their responses according to the threat level posed by the encountered microbe [1, 2]. Microorganisms captured by phagocytosis are sensed through the detection of conserved pathogen associated molecular patterns (PAMPs) by pattern-recognition receptors (PRRs) [3]. Transmembrane PRRs such as toll-like receptors (TLRs) localize to the cell surface, endosomes and phagosomes and signal the production of pro-inflammatory cytokines such as TNFα, IL-6 and IL-12, as well as chemokines and factors that promote T cell differentiation and activation. Other PRRs, such as nucleotide binding domain leucine rich repeat containing proteins (NLRs), localize to the cytosol, sense phagosome/lysosome damage [4], and signal the production of more potent pro-inflammatory cytokines such as IL-1β and IL-18 [5, 6]. Importantly, TLR and NLR responses are collaborative. Signaling by TLRs or related receptors at the plasma membrane or on phagosomes “primes” inflammasomes–multisubunit complexes that incorporate NLRs–by stimulating transcription of some NLRs and pro-IL-1β [7]. Subsequent stimuli trigger inflammasome assembly, with consequent proteolytic cleavage of pro-IL-1β and of constitutively expressed pro-IL-18, and secretion of the mature cytokines by undefined mechanisms [8, 9]. In some cases inflammasome activation leads to pyroptotic host cell death, thus restricting intracellular pathogen replication [10]. Other than transcriptional priming, the molecular links between TLR activation in phagosomes and inflammasome activity in the cytosol in response to bacterial pathogens are unclear [11].

Phagosome dynamics might not only regulate inflammasome activation but also inflammasome silencing to limit tissue damage and prevent chronic inflammation [12]. Among the identified negative regulators of inflammasome activity is autophagy [13, 14], in which cellular components are engulfed by and sequestered into double-membrane compartments (autophagosomes) and may be degraded upon autophagosome fusion with lysosomes. Intriguingly, autophagy is rapidly induced following phagocytosis of bacterial pathogens such as *Listeria* or *Salmonella* Typhimurium [15, 16, 17]. Thus, signaling from maturing phagosomes could potentially limit the duration of inflammasome activation through autophagy. How this is integrated at the molecular level is largely unknown.

We have shown that in murine DCs, adaptor protein-3 (AP-3)–an endosomal adaptor protein complex that facilitates cargo sorting into transport vesicles–optimizes the recruitment of TLRs from endosomes to maturing phagosomes and is required for efficient pro-inflammatory TLR signaling and antigen presentation from phagosomes [18]. Here we tested whether AP-3 also plays a role in inflammasome assembly and activation and subsequent T cell responses by assessing whether these processes are impaired in AP-3 deficient mice. We show that AP-3 is required in DCs for optimal inflammasome activity triggered by particulate stimuli *in vitro* and *in vivo* by three distinct mechanisms: AP-3 promotes inflammasome priming, spatially regulates inflammasome assembly and protects inflammasomes from autophagy. Our data provide new insights into the mechanisms underlying the recurrent bacterial infections in patients with mutations in AP-3 subunit genes [19, 20], and suggest that AP-3 is a key regulator that links phagosome signaling to a sustained inflammasome response to intracellular pathogens.

## Results

### AP-3 is required for optimal transcriptional activation of pro-IL-1β and some NLRs after priming with particulate LPS

**Fig 1:**
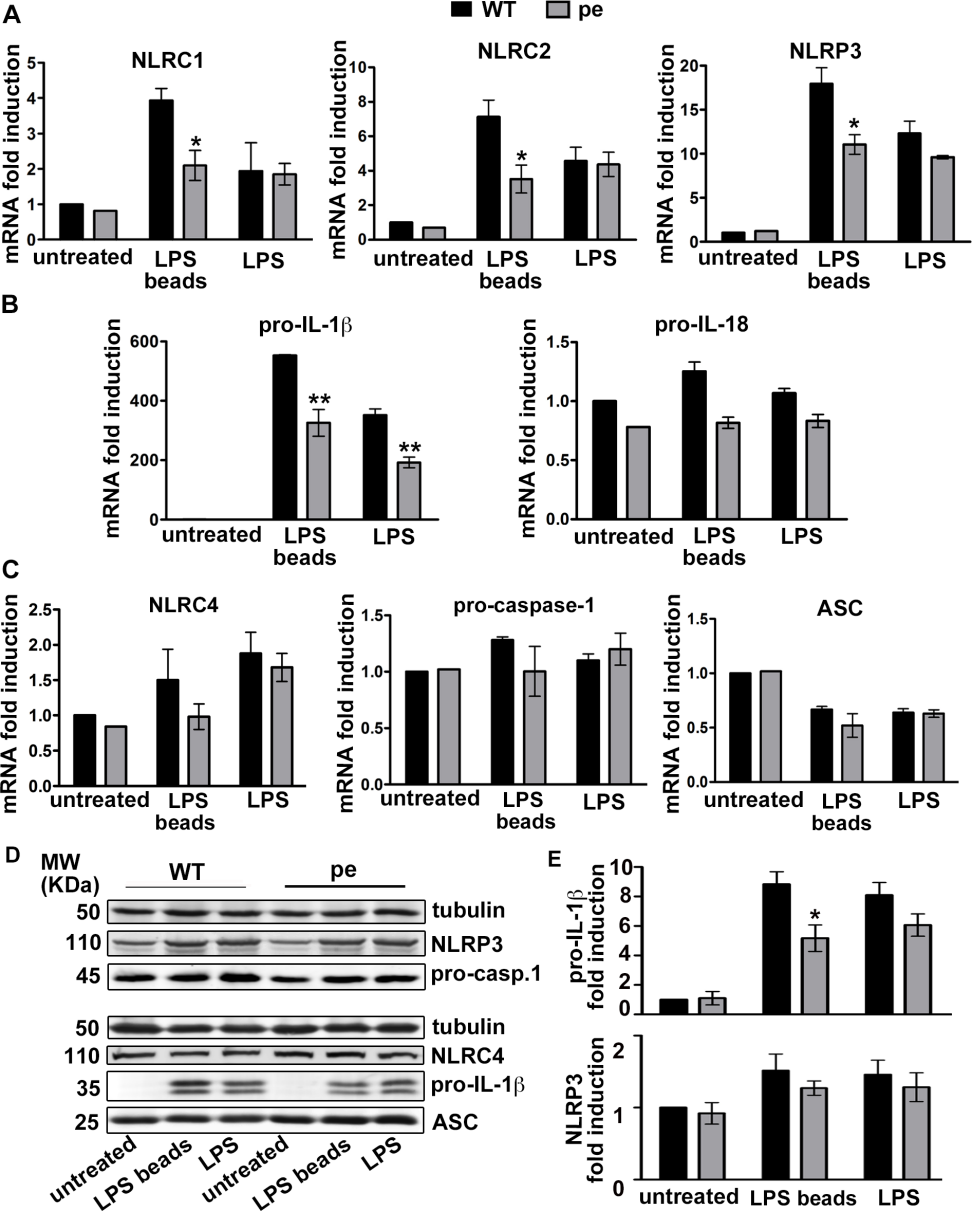
AP-3 is required for optimal transcriptional activation of pro-IL-1β and some NLRs after particulate LPS priming. BMDCs from WT or pearl (pe) mice were untreated or stimulated with LPS or LPS beads for 2 h (**A**-**C**) or 3 h (**D**, **E**). **A-C**. cDNA generated from isolated RNA was analyzed by RT-PCR. Shown are mRNA levels of: **A**, NLRC1, NLRC2, NLRP3; **B**, pro-IL-1β, pro-IL-18; and **C**, NLRC4, pro-caspase-1 and ASC. Data from three independent experiments were normalized to the average of five housekeeping genes, and the ΔΔCt values were calculated and represented as mean ± SD fold induction of mRNA in stimulated cells relative to unstimulated cells. **D**, **E.** Cell pellets were lysed and fractionated by SDS-PAGE, and NLRP3, NLRC4, pro-caspase-1 (pro-casp. 1), pro-IL-1β and ASC were detected by immunoblotting. **D**, representative blots. **E**, quantification of band intensities represented as mean ± SD fold induction in stimulated cells relative to unstimulated cells for pro-IL-1β (*top*) and NLRP3 (*bottom*), normalized to tubulin levels. Two or more fold induction was considered significant. *p< 0.05; **p<0.01. See also **S1 Table**.

AP-3 is required in DCs for optimal TLR recruitment to phagosomes and subsequent phagosomal pro-inflammatory signaling, but not for TLR signaling from the plasma membrane [18]. Because inflammasome priming requires PRR signaling [8], we investigated whether AP-3 impacts inflammasome priming by soluble LPS (to stimulate cell surface TLR4) or by LPS-coated beads (LPS beads; to stimulate phagosomal TLR4). We stimulated bone marrow-derived DCs (BMDCs) from wild-type (WT) C57BL/6J or AP-3-deficient B6.Ap3b1^*pe/pe*^ (pearl) mice with TLR4 stimuli, and measured mRNA levels for NLR and/or inflammasome pathway components by quantitative real-time (RT)-PCR (qPCR). In response to soluble LPS, mRNA levels for the NLR members NLRC1, NLRC2 and NLRP3 were induced 2- to 12-fold, with no significant differences between WT and pearl DCs (**Fig 1A**). However, the induction of mRNA for all three NLR members in response to LPS beads was 50% lower in pearl DCs than in WT DCs (**Fig 1A**; **S1 Table** provides data for all analyzed mRNAs). This is consistent with the known role of AP-3 in phagosomal TLR signaling. Transcriptional activation of the pro-inflammatory cytokine precursor pro-IL1β was also reduced in pearl DCs, but surprisingly in response to either soluble LPS or LPS beads (**Fig 1B**), suggesting additional complexity in IL-1β transcriptional control; despite this effect of soluble LPS on mRNA levels, pro-IL-1β protein levels were stimulated to significantly reduced levels in pearl DCs only after particulate LPS treatment (by ~50%; **Fig 1D and 1E**). In contrast to the strong induction of pro-IL-1β, NLRP3 induction by either soluble or particulate LPS was moderate, consistent with previous reports [21], and not significantly affected at the protein level in pearl cells (**Fig 1A, 1D and 1E**). Consistent with their known constitutive expression [4, 8, 9], mRNA levels for pro-IL-18 (**Fig 1B**) and mRNA and protein levels for NLRC4, pro-caspase-1, and the adaptor protein ASC (**Fig 1C and 1D**) were neither significantly different between WT and pearl DCs nor significantly induced after soluble or particulate LPS priming (**Fig 1B and 1C**).

### AP-3 promotes inflammasome activation by particulate stimuli

**Fig 2:**
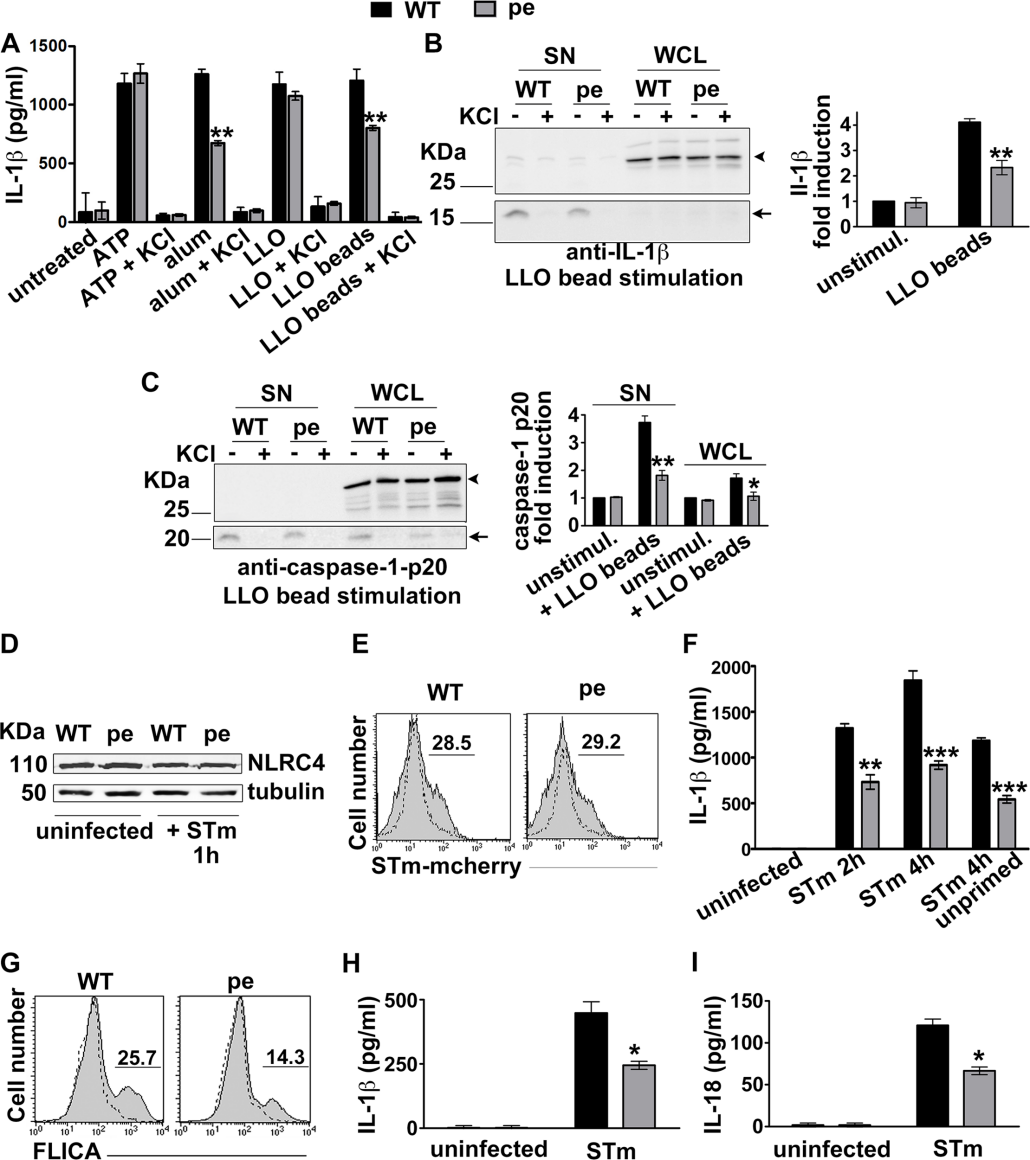
AP-3 promotes inflammasome activation triggered by phagocytosed stimuli. **A**-**C**. BMDCs from WT or pearl (pe) mice were primed for 3 h with LPS and stimulated with the indicated NLRP3 inflammasome stimuli. After 4 h cell supernatants were collected and cell pellets were lysed and fractionated by SDS-PAGE. **A**. IL-1β was detected by ELISA on cell supernatants. **B**, **C**. pro-IL-1β and mature IL-1β (**B**) or caspase-1 and its p20 cleavage fragment (**C**) were detected by immunoblotting on both supernatants and cell lysates. *Left*, representative blots. *Right*, quantification of band intensities for mature IL-1β (**B**) or caspase-1 p20 (**C**) from three independent experiments, showing fold increase relative to unstimulated cells and normalized to whole cell lysates (mean ± SD). **D-I**. BMDCs (**D-G**) or splenic DCs (**H**, **I**) were uninfected or infected with flagellin-expressing STm (stimulates NLRC4) at 10 MOI. **D.** Representative immunoblot showing NLRC4 expression and tubulin as loading control. **E**. Uninfected cells (dotted lines) or cells infected for 15 min with mCherry-labeled STm (STm-mCherry; gray filled lines) were washed and analyzed by flow cytometry. Shown is a representative of 3 independent experiments. Numbers represent percent of cells with above background mCherry signal. **F**. At the indicated time points after infection with STm, cell supernatants were collected and assayed for IL-1β by ELISA. **G.** The caspase-1 probe FAM-FLICA was added after STm infection. After 15 min, cells were washed and analyzed by flow cytometry. Dotted lines, uninfected cells; gray filled lines, infected cells. Shown is a representative experiment. **H, I.** Supernatants from splenic DCs that were untreated or infected for 4 h with STm were collected and assayed for IL-1β (**H**) or IL-18 (**I**) by ELISA. Data in **D**-**I** are from three independent experiments; data in **F**, **H** and **I** show mean ± SD. *p<0.05; **p<0.01; ***p<0.001.

To test whether AP-3 influences inflammasome activation as well as priming, we assessed IL-1β and IL-18 secretion by WT and pearl DCs in response to soluble or phagocytosed inflammasome stimuli. We pre-treated BMDCs with soluble LPS, which induced NLR transcription to the same extent in both WT and pearl DCs (**Fig 1**), and then triggered the NLRP3 inflammasome with either a soluble stimulus (ATP or the recombinant pore forming lysteriolysin O toxin (LLO) or particulate stimuli [alum or polystyrene beads coated with LLO (LLO beads)]. KCl was added in control samples to inhibit inflammasome activation after priming [22]. Whereas KCl-sensitive IL-1β secretion (measured by ELISA of cell supernatants) stimulated by soluble ATP or LLO was similar from WT and pearl DCs, IL-1β secretion in response to the particulate stimuli was reduced by ~50% in pearl DCs relative to WT cells (**Fig 2A**). Reduced IL-1β secretion by pearl DCs in response to LLO beads was confirmed by Western blotting of cell supernatants (SN, arrows; **Fig 2B**), whereas LPS-stimulated pro-IL-1β levels in pearl DC whole cell lysates (WCL) were not reduced as much relative to WT DCs (arrowheads, **Fig 2B**; see also **Fig 1D** and **1E**). These data suggest that AP-3 potentiates inflammasome activity induced by phagocytosed stimuli. Consistently, analysis of the caspase-1 p20 cleavage fragment in both cell lysates and supernatants showed that pearl DCs undergo ~50% less pro-caspase-1 cleavage than WT DCs after LLO-bead stimulation (**Fig 2C**).

Since inflammasome activation *in vivo* can be triggered by pathogen invasion, we extended our analyses of inflammasome stimuli to a model intracellular bacterial pathogen, *Salmonella enterica* serovar Typhimurium (STm). STm cultured under conditions that induce flagellin expression rapidly stimulates the NLRC4 inflammasome [23], which is priming-independent and unaffected by AP-3 (**Fig 1C and 1D**). NLRC4 expression levels in WT and pearl DCs were similar both before and after STm (**Fig 2D**). Despite equivalent levels of bacterial uptake (**Fig 2E)**, pearl BMDCs secreted significantly less IL-1β than WT cells, regardless of whether or not they were previously primed with soluble LPS (**Fig 2F**). Importantly, caspase-1 activation was also reduced by ~50% in pearl DCs after STm infection, as measured by the percentage of cells labeled by the fluorescent active caspase-1 probe FAM-YVAD-FMK (FLICA; **Fig 2G**), despite no reduction in pro-caspase-1 expression in these cells (**Fig 1C** and **1D**). These data suggest that maximal inflammasome activity in response to phagocytic stimuli in BMDCs requires AP-3. To test whether this finding extends to tissue resident DCs, we monitored secretion of IL-1β and of IL-18 (which is undetectable in BMDCs) by splenic DCs after infection with flagellin-expressing STm. Like NLRC4, pro-IL-18 mRNA is constitutively expressed in DCs, as previously shown [24–26], and unaffected by AP-3 in BMDCs (**Fig 1B** and **1C**). Induced secretion of both IL-1β and IL-18 was reduced by ~50% in splenic pearl DCs relative to WT DCs (**Fig 2H** and **2I**). Together, these results indicate that AP-3 promotes inflammasome activation by phagosomal stimuli. The impaired caspase-1 cleavage and the reduced levels of secreted IL-18 after NLRC4 inflammasome stimulation suggest that AP-3 not only impacts priming but also plays a role downstream of inflammasome priming.

### AP-3 promotes survival upon *Salmonella* Typhimurium infection

**Fig 3:**
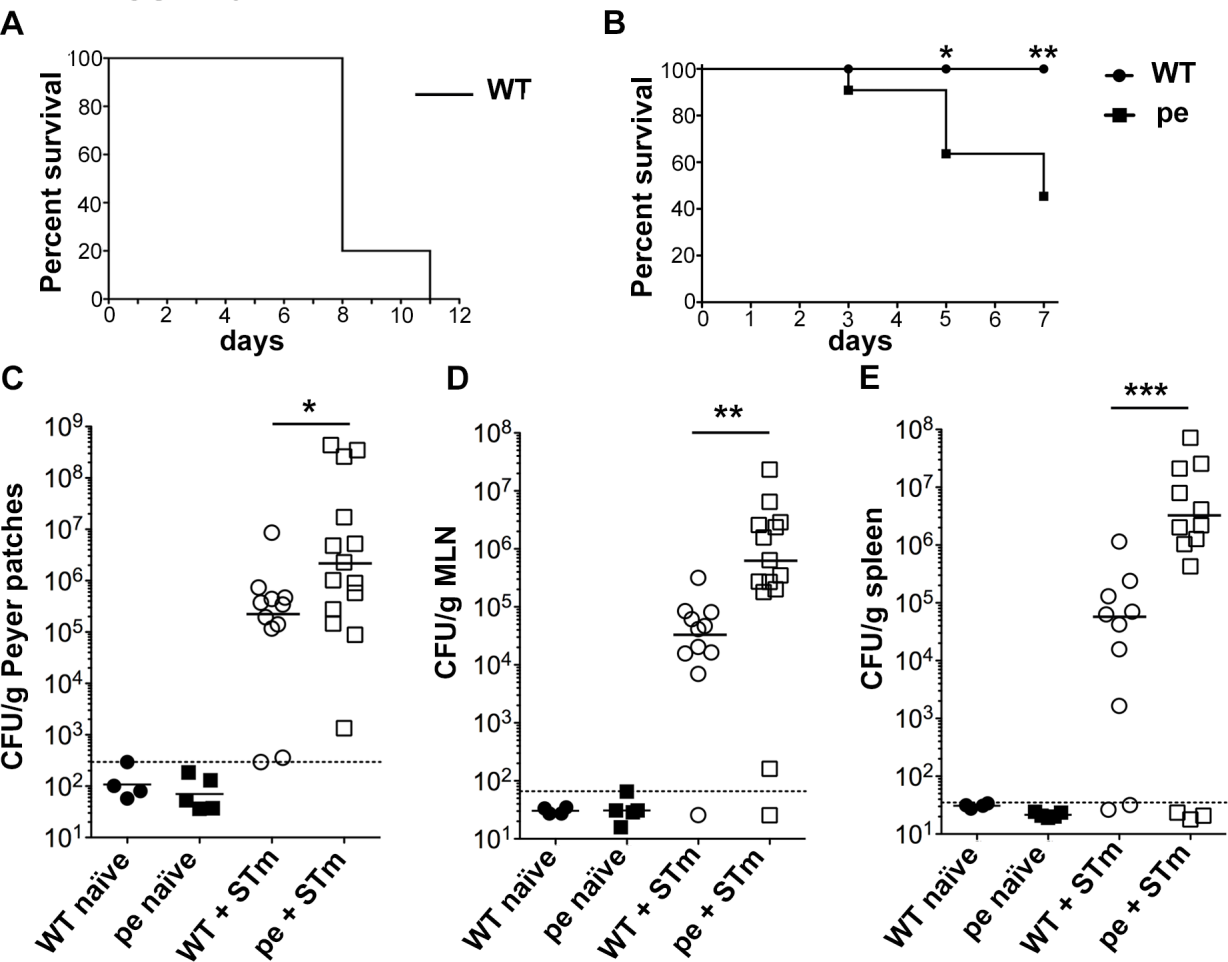
AP-3 promotes survival upon *Salmonella* Typhimurium infection. WT CD57BL/6J and congenic pearl (pe) mice were infected orally with 10^8^ STm (+ STm) or received PBS as a control (naïve). (**A, B).** Mouse survival was assessed over 12 days (**A**; n = 5) or 7 days (**B**; n = 11 each mouse type; surviving mice were euthanized on day 7). (**C-E).** Peyer patches, MLN and spleens were harvested 5 days post-infection, homogenized and plated to measure bacterial load. CFUs were normalized to tissue weight (expressed as CFU/ g of tissue). Data are pooled from three independent experiments. Dotted lines, background (threshold value from uninfected mice); solid lines, geometric means of values above background. *p<0.05; **p<0.01; ***p<0.001. See also **S1 and S2 Figs**.

To test whether AP-3 impacts inflammasome activation in DCs during an immune response, we employed an *in vivo* model of oral STm infection in mice. In this model, an early inflammatory response at the site of STm entry in the follicle-associated epithelium prevents bacterial dissemination to the bloodstream and peripheral tissues such as spleen and liver, and therefore confers protection against STm infection [27, 28]. Following infection with an oral dose of 10^8^ STm, WT mice survived to day 7 but succumb to infection later (**Fig 3A and 3B**). Under identical conditions, more than 50% of pearl mice died by day 7 (**Fig 3B**), indicating that AP-3 confers a protective response to STm infection. Analyses of colony forming units (CFU) in homogenates of Peyer's Patches (PP), mesenteric lymph nodes (MLN) and spleen harvested five days after infection showed that the bacterial burden in all three tissues was significantly higher in infected pearl mice than in WT controls (**Fig 3C**–**3E**). These data suggest that AP-3 is necessary to suppress bacterial replication *in vivo*, correlating with the increased susceptibility of AP-3-deficient patients to bacterial infections.

### AP-3 is required for optimal production of IL-1β, IL-18 and IL-17 early after STm infection

**Fig 4:**
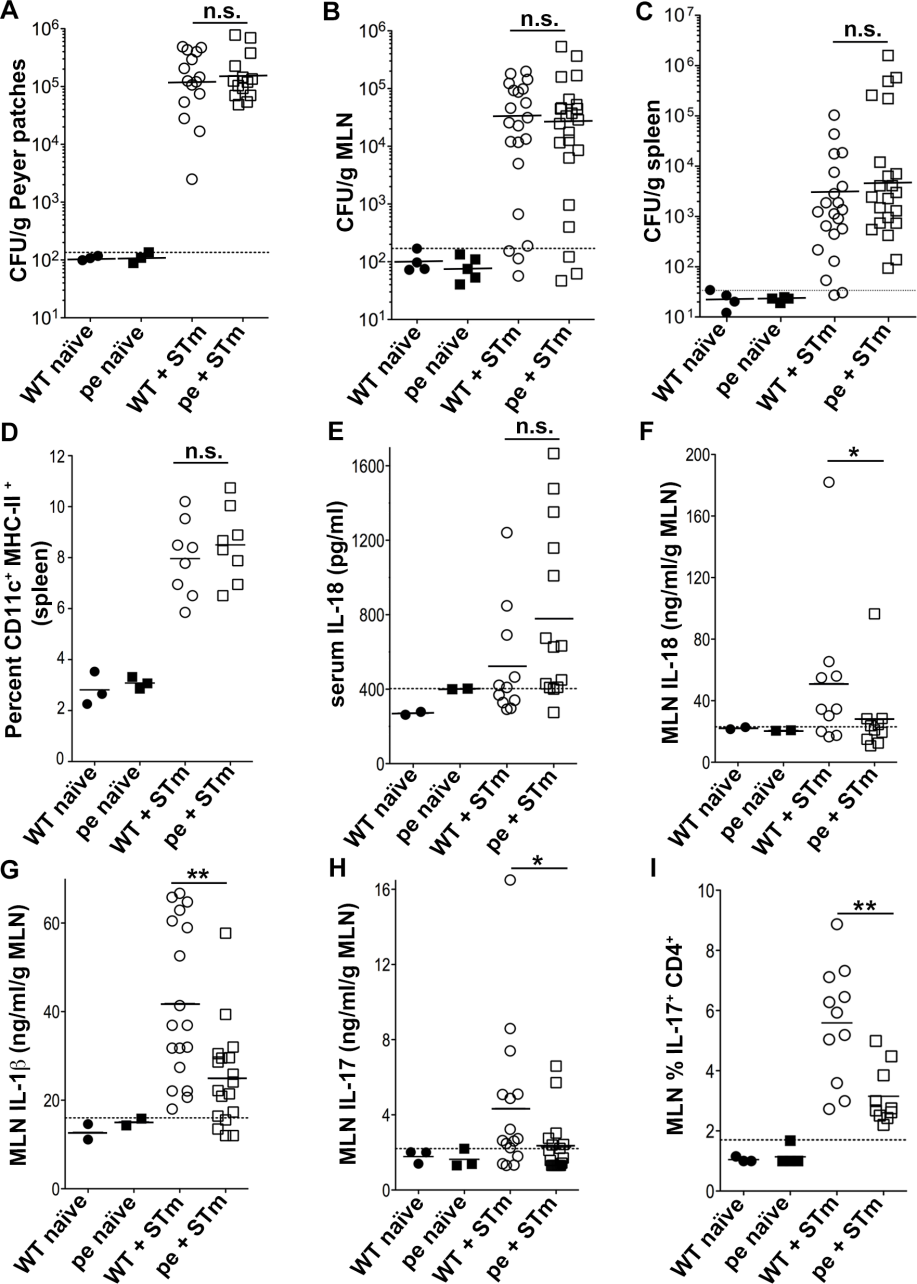
AP-3-deficient mice produce less IL-1β, IL-18 and IL-17 early after *Salmonella* Typhimurium infection. WT and pearl (pe) mice were infected orally with 10^9^ STm or received PBS as a control and analyzed three days after infection. **(A-C)**. Peyer patches, MLN and spleens were harvested, homogenized and plated to measure bacterial load. CFUs were normalized to tissue weight (expressed as CFU/ g of tissue). **D.** Cell suspensions from spleen were analyzed by flow cytometry for CD11c^+^ MHC-II^+^ DCs. Average cell number: 250x10^6^ **E.** Blood was collected by cardiac puncture, and serum was isolated and assayed for IL-18 by ELISA. (**F-H**). Supernatants from homogenized and pelleted MLN were assayed for IL-18 (**F**), IL-1β (**G**) and IL-17 (**H**) by ELISA. **I.** Single cell suspensions from MLN were restimulated *in vitro* for 5 h with 10 ng/ml PMA, 1 μg/ml ionomycin and 10 μg/ml of brefeldin A. Cells were then fixed, permeabilized, stained for intracellular IL-17 and analyzed by flow cytometry, gating on CD4^+^ T cells. Data in each panel are pooled from three independent experiments. Dotted lines, background (threshold values from uninfected mice). Solid lines, geometric mean (**A-C**) or arithmetic mean (**D-I**) of values from infected mice. *p<0.05; **p<0.01; n.s., not significant. See also **S3**and **S4 Figs**.

Intestinal DCs continuously sample the gut microenvironment and migrate from the lamina propria to MLN to maintain intestinal immune homeostasis. Upon oral infection with STm, DCs capture invading STm within the subepithelial dome of the follicle-associated epithelium and then migrate to PP and/or MLN, where they stimulate antigen-specific T cells [28, 29]. Early production of local IL-1β in the MLN and PP promotes the differentiation of Th17 cells, and subsequent IL-17 secretion by T cells and innate lymphoid cells favors granulopoiesis and neutrophil recruitment [30]. To test whether AP-3 plays a role in these early events, we compared the inflammatory cytokine responses to STm infection in WT and pearl mice. We adjusted the infection dose (to 10^9^ CFU) to achieve comparable levels of bacterial load in PP, MLN and spleen three days after infection of WT and pearl mice (**Fig 4A–4C**). At this time and dose, DC numbers in spleen (the largest lymphatic organ and hence preferred for the assessment of cell populations) of WT and pearl mice were also comparable (**Fig 4D**), suggesting that DC migration was not affected. Similarly, DC numbers in spleen, MLN and small intestine were also comparable at steady-state (uninfected) conditions in WT and pearl mice (**S1A** and **S1B Fig** and ref. [18]). Importantly, the capacity of WT and pearl CD11c^+^ DCs to mature (assessed by up-regulation of the costimulatory molecules CD40 and CD86) and their migratory potential (assessed by up-regulation of CCR7, responsible for DC homing to T cell areas of lymph nodes and spleen; [31, 32]) in response to STm infection *ex vivo* did not significantly differ (**S1C** and **S1D Fig** and ref. [18]), in agreement with the similar DC percentages in those tissues.

We then used ELISA to measure IL-1β, IL-18 and IL-17 levels in serum and/or tissue homogenates of mice 3 days after infection with 10^9^ STm. Whereas serum IL-18 levels–reflecting contributions from many infected cell types (see below)–did not significantly differ between WT and pearl mice (**Fig 4E**; see below), IL-18 levels in MLN from pearl mice were significantly reduced (**Fig 4F**). Similarly, IL-1β levels in MLN were significantly decreased in pearl mice (**Fig 4G;** IL-1β was not detected in PP or serum from infected mice). These results suggest that MLN are the key sites for early inflammatory responses to STm and that these responses are defective in the absence of AP-3, reflecting a failure in local inflammasome activation. Consistent with an IL-1β requirement in lymphocyte IL-17 production [33], IL-17 levels were also significantly decreased in MLN from pearl mice (**Fig 4H**), correlating with a reduced percentage of IL-17-producing CD4+ T cells in the MLN (**Fig 4I**). Together, these data show that inflammasome activation and IL-17 production at STm entry sites are impaired in the absence of AP-3 and likely explain, at least in part, the reduced viability of pearl mice upon STm infection.

### Inflammasome activation is impaired in AP-3-deficient dendritic cells but not macrophages

It was surprising that serum IL-18 levels in STm-infected pearl mice were high at day 3 (**Fig 4E**) despite the reduced IL-18 production in MLN relative to infected WT mice. Moreover, five days after infection with 10^8^ CFU of STm, cytokine levels in pearl and WT MLN were similar (despite higher bacterial burden in pearl MLN), while serum Il-18 levels were higher in pearl than in WT mice correlating with the increased bacterial load (**Figs 3B–3D and S2**). Serum cytokine levels reflect the contribution of different tissues and cell types, and although DCs capture STm early after infection and play a major role in the subsequent immune response [29], macrophages and other cell types also produce IL-1β and IL-18 and likely predominate cytokine production later after infection. We thus wondered whether AP-3 impacted cytokine production in another cell type. Indeed, whereas cultured pearl BMDCs secreted ~50% less IL-1β than WT DCs in response to STm infection, there was no impairment in STm-stimulated IL-1β secretion by bone marrow-derived macrophages (BMMΦs) from pearl mice (**S3A Fig**). These data indicate that AP-3 does not play a role in inflammasome activation in MΦs. Similarly, AP-3 does not affect phagosomal TLR signaling in MΦs as it does in DCs, as secretion of the pro-inflammatory cytokines TNFα, IL-6 and IL-12 in response to LPS beads is defective in pearl DCs [18] but not in pearl MΦs (**S4 Fig**); this likely reflects differences in TLR signaling requirements and inflammasome activation between DCs and MΦs [34].

To test whether AP-3 plays a role in phagosome TLR signaling and inflammasome activation in MΦs *in vivo*, we exploited the intracellular pathogen *Legionella pneumophila* (Lpm). Alveolar macrophages are the primary cell type that supports Lpm replication during pulmonary infection in mice [35]. We infected WT and pearl mice intranasally with Lpm strain Δ*flaA*, which lacks flagellin and stimulates the NLRP3 inflammasome [36, 37], and 24 h post-infection measured bacterial load in lung homogenates and levels of TNFα (as a read-out for effective priming) and IL-18 (as a read-out for inflammasome activation) in the broncho-alveolar lavage (BAL). Unlike for STm, bacterial burden was not significantly higher in pearl mice relative to WT mice (**S3B Fig**). Moreover, both TNFα and IL-18 levels were similar in BAL from WT and pearl mice (**S3C** and **S3D Fig**). These data support the conclusion that AP-3 does not play a role in inflammasome priming or activation in MΦs either *in vitro* or *in vivo*, and suggest that AP-3 impacts the inflammatory response to bacterial infections primarily via DCs.

### AP-3 is required for inflammasome positioning and limits autophagy induction after *S*Tm infection or alum stimulation

**Fig 5:**
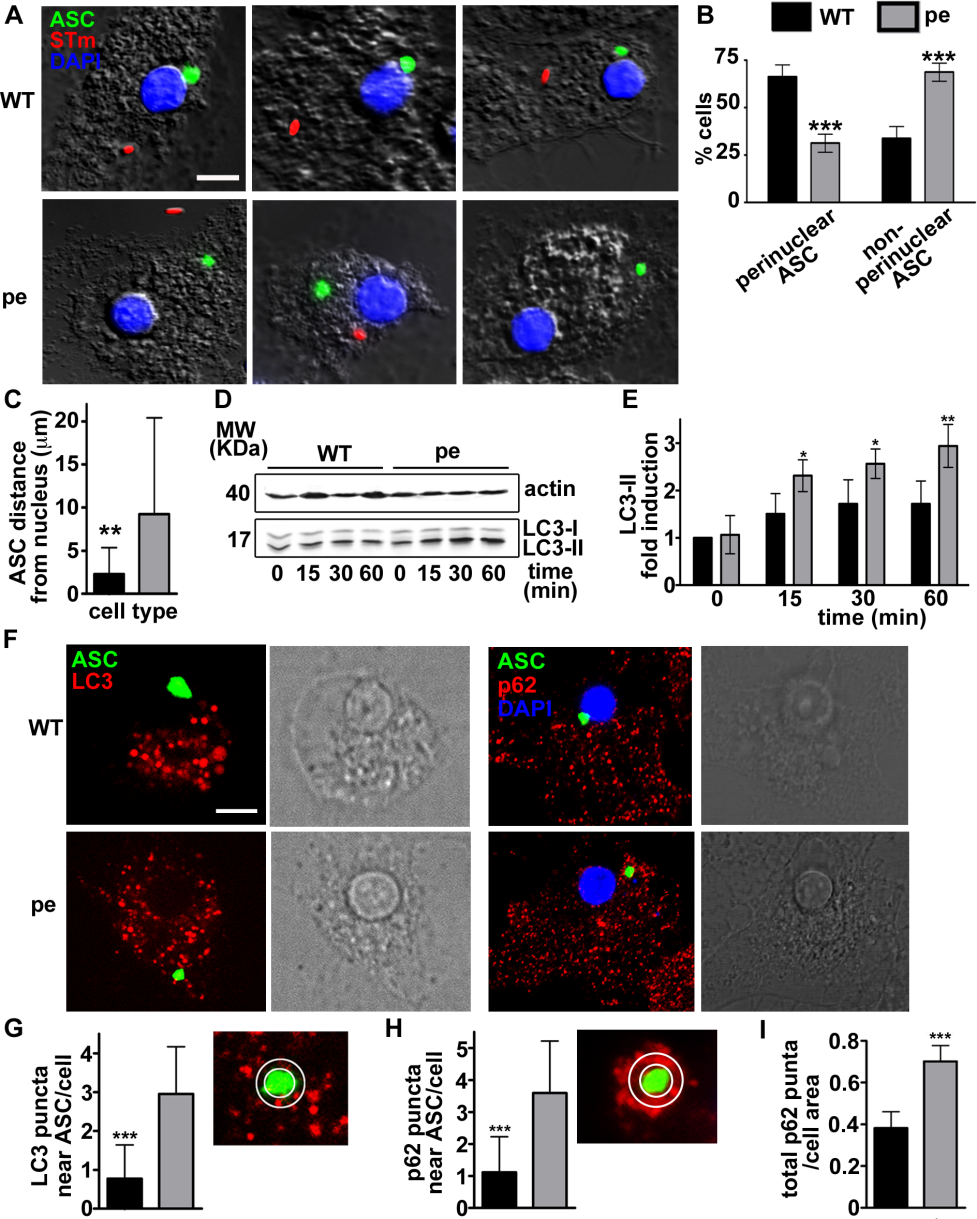
AP-3 is required for perinuclear inflammasome positioning and limits autophagy induction after *Salmonella* Typhimurium infection in DCs. **(A-C).** WT and pearl (pe) BMDCs expressing ASC-GFP were infected with flagellin-expressing mCherry-STm (stimulates NLRC4). Cells were fixed 1 h after infection, labeled with DAPI and analyzed by fluorescence microscopy. **A.** Representative images showing ASC speck (green) relative to STm (red) and nucleus (blue) in four infected WT and pearl DCs each. **B.** Quantification of perinuclear (within a radius of one μm from the nucleus) and non-perinuclear ASC specks in 20 cells per cell type in each of four independent experiments. **C.** Quantification of ASC speck distance to the nucleus in 15 cells per cell type in each of three independent experiments. **(D**, **E)**. WT and pearl BMDCs were infected with STm, and endogenous LC3 (and actin as a control) was detected by immunoblotting fractionated cell lysates at the indicated time points. **D.** Representative blot with positions of molecular weight markers (MW) indicated at left. **E**. Quantification of LC3-II band intensities from three independent experiments, expressed as fold increase relative to unstimulated cells and normalized to LC3-I and β-actin levels. (**F**-**I).** WT and pearl BMDCs expressing ASC-GFP alone or with mCherry-LC3 were infected with STm and analyzed by live fluorescence imaging (for LC3) or fixed immunofluorescence microscopy (for p62) 1 h later. **F.** Representative images showing ASC speck (green) and either LC3 puncta (red, *left panels*) or endogenous p62 puncta (red) and nuclei (blue; *right panels*) in infected cells. Corresponding DIC images show nuclear position. **G, H.** Quantification of LC3 (**G**) or p62 (**H**) puncta within a radius of 0.5 μm from the ASC speck (representative image shown at right) in 15 cells per cell type in each of 3 independent experiments. **I**, Quantification of total p62 puncta normalized to cell area. Data represent mean ± SD. Scale bar: 10 μm.**p<0.01; ***p<0.001. See also **S5**and **S6 Figs**.

AP-3 is well known to regulate protein trafficking within the endolysosomal and phagosomal systems [38], but how might it impact activity of the cytoplasmic inflammasome? Given that AP-3 influences the positioning of lysosomes and lysosome-related organelles [39, 40], we speculated that AP-3 might indirectly influence inflammasome activity via its positioning and/or assembly. Thus, we assessed the assembly of the adaptor protein apoptosis-associated speck-like protein containing a caspase-recruitment domain (ASC) into perinuclear filamentous aggregates, or “specks”, upon inflammasome activation [41, 42]. BMDCs expressing ASC-GFP from a recombinant retrovirus were infected with flagellin-expressing mCherry-STm to stimulate NLRC4, and ASC speck formation was visualized by live cell imaging. In both WT and pearl DCs, ASC-GFP was expressed diffusely in the cytoplasm prior to infection (**S5A Fig**), but formed a single speck within 15 min following STm infection, as expected upon inflammasome activation [43]. Neither the number of ASC speck-positive DCs nor the kinetics of ASC speck formation after STm infection differed appreciably between WT and pearl cells (**S5B Fig**), and in neither case did specks overlap significantly with STm-labeled structures (**Fig 5A**). Consistent with previous reports [44], ASC specks were detected adjacent to the nucleus in ~70% of WT DCs one hour after infection, such that the average distance from the nuclear membrane was 2.3 ± 3 μm and all specks were within 7 μm (**Fig 5A**–**5C**). Surprisingly, however, few ASC specks (~30%) were detected in the perinuclear area of pearl DCs (**Fig 5A** and **5B**), and the average distance to the nuclear membrane was 9.2 ± 11 μm (**Fig 5C**). ASC specks were also predominantly peripherally positioned when ATP or alum were used to trigger inflammasomes in DCs (**S5C Fig**), and even when BMMΦs were infected with STm or stimulated with alum (**S5D and S5E Fig**). This suggests that altered inflammasome positioning is a general property of AP-3-deficiency and is not unique to DCs.

We hypothesized that peripheral positioning in pearl DCs could facilitate premature inflammasome inactivation following STm infection, perhaps via autophagy. Autophagy limits AIM-2 inflammasome activation via ASC ubiquitylation and engagement of the autophagic adaptor p62/SQTM1 (p62) and the microtubule-associated protein 1 light chain 3 α (LC3) [14], and has been implicated in limiting NLRP3 activation [45–47]. As described [15], STm infection induced an early autophagic response in WT DCs and MΦs, as shown by induction of the active lipidated form of LC3 (LC3-II) relative to the unlipidated LC3-I form in cell lysates following infection (**Figs** **5D**, **5E**, **S6F** and **S6G**). Intriguingly, LC3-II activation was more rapid and more robust in pearl DCs relative to WT DCs following STm infection (**Fig 5D and 5E**) or alum stimulation (**S6A and S6B Fig**; note, LC3-II induction in WT DCs in response to alum varied among experiments, but was always less than in pearl DCs), but not in pearl MΦs relative to WT MΦs (**S6F–S6I Fig**). These data indicate that AP-3 limits autophagy specifically in DCs. Accordingly, the number of punctate structures labeled by endogenous p62 per unit area was significantly increased in pearl DCs compared to WT after STm infection (**Fig 5I**). In contrast, the induction of LC3-II in response to ATP stimulation did not differ between WT or pearl DCs (**S6C and S6D Fig**; note, LC3-II induction in response to ATP varied among experiments, but was always similar in WT and pearl DCs), correlating with the lack of impact on inflammasome activity.

To determine whether increased autophagy in pearl DCs might hasten inflammasome inactivation, we first tested whether ASC specks were detected in proximity to p62 or activated LC3. WT or pearl BMDCs expressing ASC-GFP and LC3-mCherry were infected with STm and analyzed one h later by live cell microscopy. In most WT DCs, LC3 puncta were excluded from a zone surrounding the perinuclear ASC specks (**Figs 5F** and **S6E**), suggesting that inflammasomes were protected from autophagy. In contrast, LC3 puncta were abundant in the more distal region in which ASC specks were detected in most pearl DCs (**Figs 5F** and **S6E**), such that significantly more LC3 puncta were detected within a 0.5 μm radius surrounding the ASC speck in pearl DCs (3.6 ± 1.6) than in WT DCs (1.1 ± 1.1) (**Fig 5G**). Similarly, p62 overlapped significantly more with ASC in ASC-GFP-expressing pearl DCs than in WT DCs after STm infection (**Fig 5F, 5H and S6E**). This suggested that peripheral inflammasome specks were more accessible to autophagosomes in pearl DCs.

**Fig 6:**
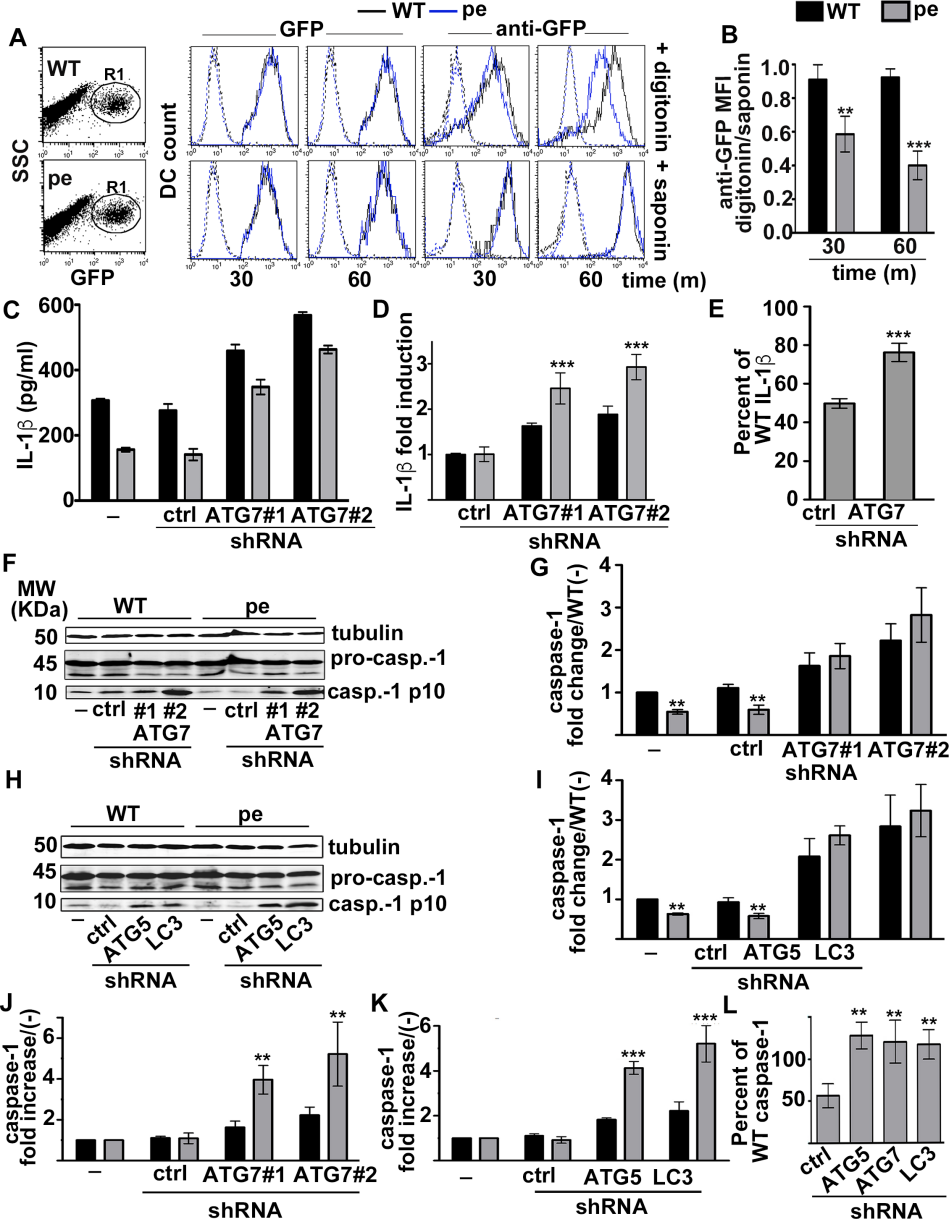
AP-3 limits inflammasome sequestration and autophagy induction after STm infection or alum stimulation. **(A**, **B)**. WT and pearl (pe) BMDCs expressing ASC-GFP were infected with flagellin-expressing STm (stimulates NLRC4) for 30 or 60 min. Cells were then permeabilized for 1 min with 50 μg/ml digitonin or throughout labeling with 0.1% saponin as indicated, washed, and incubated with mouse anti-GFP and allophycocyanin (APC)-conjugated anti-mouse antibodies. Cells were analyzed by flow cytometry, gating on GFP^+^ cells (R1). **A**. Shown are representative dot plots of transduced WT and pe DCs indicating gated region based on GFP fluorescence and side scatter (SSC, *left panels*), and representative histogram plots indicating GFP (*middle panels*) or APC (anti-GFP) fluorescence (*right panels*). *Black lines*, WT; *blue lines*, pe; *dotted lines*, secondary antibody alone. **B**. The ratio of mean fluorescence intensity (MFI) values for anti-GFP signal in digitonin-treated DCs relative to saponin-treated DCs is shown from 4 independent experiments. (**C-L)**. WT and pearl (pe) BMDCs that were non-transduced (-) or transduced with lentiviruses encoding non-target (ctrl) or either of two ATG7-specific shRNAs or ATG-5- or LC3b-specific shRNAs were infected with STm (**C-E**) or primed for 3 h with soluble LPS and stimulated with alum (**F-L**). (**C-E**) Cell supernatants collected 2 h after Stm infection were assayed for IL-1β by ELISA. **C.** Representative experiment. **D**. Data from 3 independent experiments were normalized to IL-1β values from cells treated with non-target shRNA and presented as fold induction. **E**. IL-1β values for pearl BMDC treated with non-target or ATG7 shRNAs from 3 independent experiments are shown as percent of values for WT DCs treated with the same shRNAs. (**F-L**) Cell pellets collected 4 h after alum stimulation were lysed, fractionated by SDS-PAGE and immunoblotted for caspase-1 and tubulin. (**F, H**). Representative immunoblots, showing pro-caspase-1 (pro-casp.-1) and mature p10 (casp.-1 p10) bands. (**G, I**) Quantification of band intensities for caspase-1 p10 normalized to pro-caspase-1 and tubulin from three independent experiments are shown as caspase-1 fold change relative to unstimulated (-) WT cells (mean ± SD). (**J, K**) Data from three independent experiments were normalized to caspase-1 values from untreated cells and presented as fold increase. (**L**). Caspase-1 values for pearl BMDC treated with non-target, ATG5, ATG7 or LC3b shRNAs from 3 independent experiments are shown as percent of values for WT DCs treated with the same shRNAs. Data in all panels represent mean ± SD. **p<0.01; ***p<0.001. See also **S7 Fig**.

We thus considered whether inflammasome fragments might become sequestered in autophagosomal membranes. To determine whether inflammasomes become sequestered by autophagosomes more in pearl DCs than in WT DCs, we used flow cytometry to quantify the accessibility of ASC-GFP to anti-GFP antibodies in permeabilized STm-infected cells. We exploited the different lipid composition of the plasma membrane relative to intracellular membranes, and either selectively permeabilized the plasma membrane by brief exposure to a low concentration of digitonin or permeabilized all membranes with 0.1% saponin [48]. These treatments were effective, since the cytosolic domain of the endoplasmic reticulum (ER) transmembrane protein calnexin, but not the ER luminal enzyme protein disulfide isomerase (PDI), was detected in digitonin-treated cells, whereas PDI was detected in saponin-treated cells (**S7A Fig**). The level of GFP fluorescence from ASC-GFP was identical in transduced WT and pearl DCs using either treatment (**Fig 6A**, *left and middle panels*), and although anti-GFP fluorescence qualitatively overlapped with ASC-GFP in both WT and pearl DCs by microscopy (**S7B Fig**), quantification showed that the anti-GFP fluorescence intensity decreased over time only in pearl DCs treated with digitonin (**Fig 6A**, *top right panels*); this quantitative decrease was ablated in cells treated with saponin (**Fig 6A**, *bottom right panels*). These data indicate that ASC becomes inaccessible to cytoplasmic antibody over time after STm infection more in pearl DCs than in WT DCs, consistent with sequestration of inflammasome fragments by autophagic membranes.

To test whether sequestration of inflammasome fragments by membranes and proximity to LC3 and p62 reflected inflammasome inactivation by autophagy, we assessed the consequences of impairing autophagy upon silencing the autophagy initiator ATG7 by lentiviral expression of either of two specific shRNAs (**S7C Fig**). Silencing ATG7 did not significantly affect bacterial load or DC death during the 2h of infection with flagellin-expressing STm (**S7D and S7E Fig)**. In these conditions, STm rapidly induces pyroptosis by a mechanism that is NLRC4- and caspase-1-dependent [49, 50]. ATG7 silencing did not affect priming, as shown by the lack of effect in either WT or pearl DCs on TNFα secretion after STm infection (**S7F Fig**) or on pro-IL-1β production after stimulation with LPS-beads (**S7G** and **S7H Fig**). However, as expected [51], ATG7 silencing increased STm-induced IL-1β secretion in both WT and pearl DCs (**Fig 6C**), but to a higher degree in pearl DCs as reflected by fold increase in IL-1β secretion relative to control shRNA-transduced DCs (**Fig 6D**). Indeed, ATG7 silencing largely rescued the IL-1β secretion defect in pearl DCs to 76 ± 5% the level of ATG7-silenced WT DC levels (compared to 49 ± 2% in control shRNA-transduced cells; **Fig 6E**). Similarly, silencing the autophagy regulators ATG5 and LC3 (**S7I** and **S7J Fig**) increased alum-induced IL-1β secretion to a higher extent in pearl than in WT DCs (**S7K** and **S7L Fig**), and restored IL-1β secretion to ~ 80% of IL-β levels in similarly treated WT DCs (**S7M Fig**). The remaining IL-1β secretion defect in pearl DCs after ATG5, ATG7 or LC3 silencing likely reflects reduced pro-IL-1β levels following inflammasome priming (**Figs 1, S7G** and **S7H**); this suggests that autophagy does not limit pro-IL-1β accumulation in our brief stimulation in DCs as it does after a longer priming period in MΦs [52]. Silencing ATG5, ATG7 or LC3 also increased caspase-1 p10 production to significantly higher levels in pearl DCs than in WT cells (**Fig 6F**–**6K**), completely restoring p10 levels in pearl DCs to those observed in silenced WT DCs (**Fig 6L**). Because pro-caspase-1 expression is constitutive and unaffected by AP-3 (**Fig 1C and 1D**), this result shows that silencing autophagy restores inflammasome activity in pearl DCs.

Together, these data indicate that in the absence of AP-3, inflammasomes are more rapidly inactivated following STm infection or alum stimulation due to (1) a more robust autophagic response and (2) altered inflammasome positioning that allows greater access to the autophagic machinery and sequestration within autophagosomes.

## Discussion

The molecular processes by which phagosome maturation and membrane dynamics are linked to cytosolic inflammasome activation following bacterial pathogen uptake are incompletely understood [11]. Here we show that in DCs, the endosomal adaptor protein AP-3 facilitates inflammasome activation after phagocytosis by two distinct mechanisms. First, AP-3 promotes phagosomal TLR-dependent priming of the transcription of pro-IL-1β and NLRs such as NLRP3 [53] by optimizing TLR4 and MyD88 recruitment to phagosomes [18]. The second mechanism influences the duration of inflammasome activity, reflecting a previously unappreciated role for AP-3 in both positioning assembled inflammasome complexes at the perinuclear region and limiting the induction of autophagy by phagocytosed stimuli. Thus, peripheral inflammasomes become more rapidly inactivated by sequestration in autophagosomes in AP-3-deficient DCs. Consistent with the impaired inflammasome activity in AP-3-deficient DCs, AP-3-deficient pearl mice show a dampened inflammatory response early during oral infection with STm, characterized by decreased production of IL-1β, IL-18 and IL-17 and correlating with higher bacterial load at later time points and more rapid death. Our data provide new insights into how phagosome maturation and cytosolic signaling responses are coordinated to regulate inflammasome activity and into the etiology of the recurrent bacterial infections in AP-3-deficient patients [19, 20], and highlight new pathways by which other genetic disorders that impact autophagy might heighten or dampen proinflammatory responses.

While a requirement for AP-3 in optimal phagosome-restricted TLR-dependent priming of inflammasome components in DCs was predicted by its known role in TLR4 delivery to maturing phagosomes and in downstream proinflammatory signaling [18], the role of AP-3 as a novel regulator of inflammasome activity was surprising. Importantly, the inflammasome impairment was observed *in vitro* only after stimulation with particulate stimuli such as alum, LLO beads and STm infection and not with soluble LLO or ATP. These results–particularly the differential requirement for AP-3 in response to LLO beads vs. soluble LLO–strongly suggest that only phagosomal stimuli require AP-3 to transduce maximal inflammasome activity in DCs. The lack of an AP-3 requirement in the response to ATP might alternatively reflect phagocytosis-independent differences either in the source of radical oxygen species (ROS) required for inflammasome activation [23] or in signaling downstream of ATP-stimulated P_2_X receptors and the particulate ligands used here (as exemplified by the robust activation of autophagy by alum and STm but not by ATP). Given the recurrent bacterial infections observed in AP-3-deficient patients [19, 54] and the impaired response of pearl mice to STm infection shown here, we speculate that bacterial infections are the most physiologically relevant stimuli for which optimal inflammasome activation requires AP-3. Our data suggest that AP-3 influences silencing of the classical inflammasomes assembled with ASC, caspase-1 and either NLRC4 or NLRP3. Whether AP-3 also influences silencing of other classical inflammasomes or of non-canonical caspase-11 inflammasomes remains to be determined.

How can AP-3 regulate inflammasome activation? We propose that AP-3 prolongs inflammasome activity by delaying inflammasome inactivation through sequestration in autophagosomes using two independent but collaborating mechanisms. First, AP-3 dampens the autophagic response to phagocytosed stimuli; autophagy was induced more rapidly and to a greater degree by STm or alum, but not by ATP, in pearl DCs relative to WT DCs. Second, AP-3 regulates inflammasome positioning; whereas inflammasomes assembled rapidly into a single "speck" within the perinuclear region following STm infection in most WT cells as previously observed [55], they assembled at a distal site in most pearl cells. The perinuclear ASC specks in WT cells were inaccessible to autophagosomal membranes marked by LC3 and p62, but the peripheral specks in pearl DCs were surrounded by these structures and allowed for more rapid sequestration of inflammasomes within autophagosomes. Our model is consistent with recent reports indicating that autophagy limits the duration of inflammasome activation by targeting components such as NLRP3 and ASC for degradation [14, 56, 57], and that NLRP3 inflammasome activity in macrophages is heightened by depletion of ATG7 or p62 [58]. Our data do not rule out that autophagy might additionally limit inflammasome activity indirectly by sequestering mitochondria and preventing ROS or mitochondrial DNA release required for inflammasome activation [47]. Although LC3 and p62 closely apposed inflammasome specks in pearl DCs, complete overlap was rarely observed. Moreover, our flow cytometry analyses of ASC-GFP sequestration indicate that sequestration was incomplete, at least at the times analyzed. We interpret these data to suggest that inflammasome fragments, rather than the entire inflammasome, is sequestered within autophagosomes. This would be consistent with the large size of inflammasome specks relative to typical autophagosomal structures [59], and would suggest that the prion-like inflammasome aggregate [60] can be partially disassembled.

How AP-3 influences either inflammasome positioning or autophagy induction is not clear. AP-3 is needed to appropriately position lytic granules towards the immunological synapse in T cells [39] and to position lysosomes in HeLa and HEK293 cells [40], but these likely reflect a direct role for AP-3 in targeting receptors for microtubule motors to endolysosomal organelles [38]; the effects of AP-3 on inflammasome positioning are more likely indirect. Peripheral ASC positioning was observed in pearl DCs after treatment with ATP, which did not significantly induce autophagy, and in pearl MΦs, in which induced inflammasome activity was similar to WT MΦs, indicating that inflammasome positioning occurs independently from autophagy induction but may prepare inflammasomes for subsequent inactivation. Regardless of the mechanism, our data suggest that the perinuclear location of the ASC speck in WT cells sequesters the inflammasome from autophagic membranes, perhaps in an “exclusion zone"–a cytoplasmic region that is devoid of membranes and large cytoplasmic particles [61]. The more rapid and robust autophagic response of AP-3-deficient cells might be a consequence of AP-3's role in cargo transport to lysosomes. Given that AP-3 mediates the delivery of membrane transporters in other cell types [62, 63], we speculate that the increased autophagy in AP-3-deficient DCs reflects loss of nutrient and/or ion transporters and consequent up-regulation of the basal autophagic machinery [64]. Indeed, increased autophagy might be a general phenomenon of AP-3-deficiency in cells that exploit AP-3 for specialized trafficking processes, as AP-3-deficient melanocytes display heightened sensitivity to nutrient deprivation with features consistent with an autophagic response [62]. Our data predict that additional genetic disorders in which autophagy is upregulated will also be associated with recurrent bacterial infections by a similar mechanism.

Decreased inflammasome activity in AP-3 deficiency was observed in DCs but not in BMMΦs, mimicking differences observed between DCs and MΦs in inflammasome activation in response to microbial products, ATP and oxidized lipids [34]. This cell type-specific difference extended also to the requirement for AP-3 in maximal phagosomal TLR-induced proinflammatory cytokine secretion, and might reflect the unique phagosome maturation pathways in DCs and MΦs. Antigen degradation in DC phagosomes is limited by delayed acidification, owing in part to the activation of the NADPH oxidase complex (NOX2) [65], supporting robust antigen presentation [66]. In contrast, phagosomes acidify rapidly in macrophages to better support pathogen destruction [67]. TLR-dependent tubule formation from phagosomes is also a unique feature of DCs [68]. Perhaps most pertinent is the role of autophagy components in phagosome maturation. In our model systems, induction of LC3-II after STm infection or alum stimulation was not significantly higher in pearl MΦs compared to WT. In DCs, lipidated LC3 and other phagosome components are recruited to phagosomes and accelerate phagolysosome formation, subsequent microbial degradation, and antigen presentation [69, 70]. Autophagy components appear to function in phagosome maturation only in DCs and not in BMMΦs [71]. Although the involvement of these components in phagosome maturation does not require autophagy per se [70], the amplified activation of LC3 in AP-3-deficient cells likely influences phagosomal properties more in DCs than in MΦs.

The importance of AP-3 in optimizing DC inflammasome responses in cell culture was reflected by impaired inflammasome responses by AP-3-deficient pearl mice *in vivo*, and likely explains at least in part the increased susceptibility of AP-3-deficient patients to recurrent bacterial infections. Mutations in the gene encoding the β3A subunit ablate AP-3 function in non-neuronal cells in Hermansky-Pudlak syndrome type 2 (HPS2) [72, 73], a heritable disease characterized by immunodeficiency among other symptoms [19, 20]. Our data show that pearl mice, a model for HPS2, are more susceptible to infection with STm than control mice, resulting in higher bacterial loads in multiple tissues. Although we cannot rule out that potential microbiota differences between WT and pearl mice contribute to the enhanced susceptibility of AP-3^-/-^ mice to infection, pearl mice with a comparable STm burden at day 3 of infection had impaired IL-1β and IL-18 responses in MLN–one of the first sites of STm entry, where pro-inflammatory cytokine production is required to prevent bacterial dissemination–suggesting an intrinsic defect in inflammasome activation and consistent with our *in vitro* results in bone marrow-derived and splenic DCs. IL-17 responses were also impaired; this might be secondary to the reduced local IL-1β, since IL-1β favors the development of T helper 17 (Th17) cells [33, 74], and/or to the impaired MHC-II presentation and CD4+ T cell differentiation induced by pearl DCs [18]. Although we cannot definitively attribute the observed phenotype in STm-infected AP-3^-/-^ mice exclusively to DCs, our data suggest that macrophages do not contribute to the impaired cytokine responses, since BMMΦs from WT and pearl mice showed comparable cytokine responses and infection of WT and pearl mice with *LPm*, which primarily infects macrophages and neutrophils, did not result in a differential inflammatory response.

IL-1β and IL-17 are particularly important for the clearance of pathogens such as *Pseudomonas aeruginosa*, *Klebsiella pneumoniae*, and *Enterococci* [75, 76]. Intriguingly, HPS2 patients suffer from recurrent infections by these pathogens [54, 77], suggesting that AP-3 might support inflammasome activity and IL17-dependent pathogen clearance in humans. In addition, HPS2 patients frequently present with neutropenia [78]; although resolution of neutropenia by treatment with granulocyte colony stimulating factor does not reduce the rate of bacterial infections, neutropenia might also be a consequence of reduced IL-17 production, which is required for granulopoiesis [79]. Thus, our studies provide novel insights into the etiology of recurrent bacterial infections in HPS2 and unveil potential new targets for immunotherapy in HPS2 and other related immunodeficiencies.

## Materials and methods

### Ethics statement

Mice were bred under pathogen-free conditions in the Department of Veterinary Resources at the Children's Hospital of Philadelphia or the University Laboratory Animal Resources at the University of Pennsylvania, and were euthanized by carbon dioxide narcosis according to guidelines of the American Veterinary Medical Association Guidelines on Euthanasia. All animal studies were performed in compliance with the federal regulations set forth in the recommendations in the Public Health Service Policy on the Humane Care and Use of Laboratory Animals, the National Research Council's Guide for the Care and Use of Laboratory Animals, the National Institutes of Health Office of Laboratory Animal Welfare, the American Veterinary Medical Association Guidelines on Euthanasia, and the guidelines of the Institutional Animal Care and Use Committees of the University of Pennsylvania or Children's Hospital of Philadelphia. All protocols used in this study were approved by the Institutional Animal Care and Use Committee at the University of Pennsylvania (protocols #804714, #804928 and #803489) or at the Children’s Hospital of Philadelphia (protocols #14–001064 and #16–001064).

### Reagents

LPS purchased from InvivoGen (San Diego, CA) was coated onto 3-micron polystyrene beads (Polysciences Inc., Warrington, PA) as previously described [80]. ATP was purchased from Sigma-Aldrich (Saint Louis, MO) and alum was from ThermoScientific (Rockford, IL). The caspase-1 probe FAM-YVAD-FMK (FAM-FLICA) was from ImmunoChemistry Technologies (Bloomington, MN). Recombinant LLO was purified from *E*. *coli* strain DP-3570 expressing six histidine (His6)-tagged LLO (kindly provided by Daniel Portnoy, University of California, Berkeley), and was coated onto 3-micron polystyrene beads (Polysciences Inc.) as previously described [81, 82]. Mouse monoclonal anti-caspase-1 p20 (Casper-1), mouse monoclonal anti-caspase-1 p10 (Casper-2), mouse monoclonal anti-NLRP3 (Cryo-2) and rabbit polyclonal anti-ASC (AL177) were from Adipogen (San Diego, CA); goat polyclonal anti-IL-1β was from R&D (Minneapolis, MN); rabbit polyclonal anti-LC3B (ab48394), mouse monoclonal anti-p62 (ab56416), rabbit monoclonal anti-ATG7 (ab133528) and rabbit monoclonal anti-ATG5 (ab108327) antibodies were from Abcam (Cambridge, MA); mouse monoclonal anti-β actin and anti-γ tubulin were from Sigma; rabbit polyclonal anti-NLRC4 was from Millipore (Billerica, MA); rat monoclonal anti-CD4 (GK1.5), anti-IL-17 (TC11), anti-CD103 (M290), anti-CD40 (3/23), anti I-A^b^ (AF-120.1), anti-CD11c (HL3), anti-CD86 (GL1) and anti-CCR7 (4B12) were from BD Biosciences (San Jose, CA); mouse monoclonal anti-GFP was from Roche (Indianapolis, IN); rabbit polyclonal anti-calnexin (SPA-860) was from Stressgen Biotechnologies Corporation (Victoria, British Columbia, Canada); mouse monoclonal anti-PDI (SPA-891) was from Enzo Life Sciences (Farmingdale, NY). All secondary antibodies were from Jackson Immunoresearch (West Grove, PA) or from BD Biosciences. ELISA Ready-SET-Go! kits for mouse interleukin-6, mouse interleukin-12/IL-23 (total p40), and mouse-TNFα were from eBioscience, (San Diego, CA); the anti-mouse-IL-1β ELISA set was from BD Biosciences; anti-mouse IL-18 ELISA antibody pairs were from MBL International (Woburn, MA); and the anti-mouse IL-17 ELISA DuoSet was from R&D.

### Mice

C57BL/6 (CD45.1 or CD45.2) wild-type (WT) mice were originally purchased from The Jackson Laboratories (Bar Harbor, ME). The HPS2 mouse model pearl (AP-3 deficient; B6Pin.C3-*Ap3b1*^*pe*^/*Ap3b1*^*pe*^) was previously described [73] and kindly provided by S. Guttentag (Vanderbilt University, Nashville, TN). All mice were bred and housed in the same room to minimize possible microbiota differences. Sex- and age-matched mice between 6 and 12 weeks of age were used in all experiments.

### Cell culture

Bone marrow cells were isolated and cultured for 7–9 d in RPMI-1640 medium (Gibco, ThermoFisher, Waltham, MA) supplemented with 10% low endotoxin-FBS (Hyclone, Logan, UT), 2 mM L-Gln, 50 μM 2-mercaptoethanol (InVitrogen) and either 30% granulocyte-macrophage colony stimulating factor (GM-CSF)-containing conditioned medium from J558L cells (kindly provided by Ralph Steinman) for differentiation to DCs as described [80, 83], or 30% M-CSF-containing L929 conditioned medium for differentiation to MΦs [35]. Splenic DCs were isolated as described [84] and purified from single cell suspensions with anti-CD11c (N418) microbeads after depletion of T, B and NK cells with a cocktail of biotin-conjugated antibodies (to CD90.2, CD45R, and CD49b) and anti-biotin microbeads (Miltenyi Biotec Inc., Auburn, CA). Intestinal lamina propria was isolated after dissecting small intestines, removing Peyer’s patches and MLNs, and dissociating the intestinal epithelium in Ca^2+^/Mg^2+^-free HBSS supplemented with 5% FBS and 2 mM EDTA for 20 min at 37°. Lamina propria was then digested by treatment with 1.5 mg/ml type VII collagenase (Sigma) and 40 μg/ml DNase I (Sigma) for 15 min at 37° to obtain single cell suspensions (as shown in http://www.jove.com/video/4040/; [85]). Lamina propria cells were stained and analyzed by flow cytometry [85].

### DNA constructs, retroviral production and transduction of dendritic cells

pMSCV-ASC-GFP and pLZRS-mCherry-LC3B retroviral constructs were kindly provided by Teresa Fernandes-Alnemri (Thomas Jefferson University, Philadelphia, PA) and Erika Holzbaur (University of Pennsylvania, Philadelphia, PA) respectively. Retrovirus was produced by transfection of packaging cell line PLAT-E [86] (a generous gift of Mitchell Weiss, St. Jude Children's Research Hospital, Memphis, TN) using Lipofectamine 2000 (Invitrogen, ThermoFisher Scientific) and harvested from cell supernatants 2 d later. 3 x 10^6^ BM cells were seeded on 6-well non-tissue culture treated plates per well for transduction 2 d after isolation, and transduced by spinoculation with 3 ml of transfected Plat-E cell supernatant in the presence of 8 μg/ml polybrene and 20 mM HEPES for 2h at 37°C. Retrovirus-containing media were then replaced with DC culture media. Puromycin (2 μg/ml) or the corresponding selection antibiotic was added 3 d after infection, and cells were collected for experiments 3 d later.

### shRNAs, lentiviral production and transduction of dendritic cells

pLKO.1-puromycin derived lentiviral vectors [87] for small hairpin RNAs (shRNAs) against ATG5, ATG7, LC3b and non-target shRNAs were obtained from the High-throughput Screening Core of the University of Pennsylvania. ATG5 sense sequence: GCAGAACCATACTATTTGCTT ATG7 #1 sense sequence: GCCTGGCATTTGATAAATGTA. ATG7 #2 sense sequence: CCTAAAGAAGTACC ACTTCTA. LC3b sense sequence: GCATCCTAAGTTGCCAATAAA. Non-target sense sequence: GCGCGATAGCGCTAATAATTT. Lentivirus was produced by co-transfection of 293T cells (obtained from American Type Culture Collection, Mannassas, VA) with packaging vectors pDM2.G and pSPAx2 using calcium phosphate precipitation [88], and harvested from cell supernatants 2 d later. 3 x 10^6^ BM cells were seeded on 6-well non-tissue culture treated plates per well for transduction two d after isolation, and transduced by spinoculation with 3 ml of transfected 293T cell supernatant in the presence of 8 μg/ml polybrene and 20 mM HEPES for 2h at 37°C [84]. Lentivirus-containing media were then replaced with DC culture media. Puromycin (2 μg/ml) was added 3 d after infection, and cells were collected for experiments 3 d later.

### Bacterial strains and infections

Bacteria were grown overnight with aeration at 37°C and diluted in PBS. Mice fasted 12–16 h were orally inoculated with 10^8^ or 10^9^ STm (SL1344) as indicated or with PBS as a vehicle control. Virulence gene expression (SPI-I) is induced when STm reaches the intestine [89, 90]. Mice were sacrificed and tissues and sera were collected 3, 5 or 7 d after infection. Tissues were mechanically homogenized, diluted in PBS, and plated on streptomycin selective LB plates to determine bacterial load (CFU per gram). For *Legionella* infections, mice were anesthetized and either infected intranasally with 5 × 10^6^ *L*. *pneumophila serogroup 1* Δ*flaA* or received PBS as a vehicle control. 24 h post-infection, mice were sacrificed and bronchoalveolar lavage was performed with 1 mL cold PBS. Lungs were then excised, mechanically homogenized, diluted and plated on charcoal yeast extract agar plates to determine bacterial burden (CFU per gram).

For *in vitro* infections, STm were grown overnight in streptomycin containing LB medium at 37°C with aeration, diluted into fresh LB containing 300 mM NaCl, and grown standing at 37°C for 3 h to induce flagellin expression [23] unless otherwise indicated. Bacteria were washed with prewarmed Dulbecco’s Modified Eagle Medium (DMEM, Gibco), added to cells at a MOI of 10:1 (unless otherwise stated), and spun onto the cells at 200 x g for 5 min. Cells were incubated at 37 °C in a tissue culture incubator with 5% CO_2_. Gentamycin (100 μg/ml) was added 1 h after infection and cells were harvested or analyzed by live cell imaging at the indicated time points.

### Real-time PCR assay

RNA was isolated using RNeasy kit (Qiagen, CA, USA) from WT and pearl BMDCs after 2 h incubation with or without LPS (50 ng/ml) or LPS-coated beads. 1 μg RNA was reverse transcribed to cDNA using RT2 First Strand kit (Qiagen). Real-time PCR was performed in a 7300 ABI PCR System (Applied Biosystems, CA, USA) using RT2 SYBR Green/ROX PCR mastermix and the Mouse Antibacterial Response RT2 Profiler PCR array (96-well format, SABiosciences, Qiagen). Data were normalized to the average of five housekeeping genes (β-glucuronidase, hypoxanthine guanine phosphoribosyl transferase, heat shock protein 90α, glyceraldehyde-3-phosphate dehydrogenase and β-actin). Relative levels of IL6, IL12p35, IL12p40 and IFNβ1 mRNA were calculated with the ΔΔCt method [18] and represented as mRNA fold-induction compared to unstimulated cells. Only two-fold or more differences were considered significant, according to manufacturer’s instructions (Qiagen).

### Inflammasome activation and measurement of cytokine production

200,000 BMDCs or BMMΦs were seeded in triplicate in RPMI medium on 96-well round bottom plates, primed where indicated with 20 ng/ml of soluble LPS for 2–3 h, and then incubated with the indicated inflammasome stimuli (1 mM ATP, 100 μg/ml alum, 10 μg/ml LLO, 10 x 10^6^ LLO beads or *Salmonella* at MOI of 10:1), for 1 to 4 h. 50 mM KCl was added to some wells for 1 h before stimulus addition to inhibit inflammasome activation [91]. Cells were pelleted at 200 x g at the indicated time points, supernatants were collected to measure cytokines using commercial ELISA kits, and cell pellets were lysed with Laemmli sample buffer with 2-mercapto-ethanol for immunoblotting analysis [91]. To detect caspase-1 activity by flow cytometry, BMDCs were infected with *Salmonella* as described or left untreated, incubated with caspase-1 probe FAM-FLICA according to the manufacturer’s instructions, washed with PBS, and analyzed using a FACSCalibur and CellQuest software (BD Biosciences).

For cytokine detection after *in vivo* infections, tissue homogenates were pelleted and supernatants were analyzed using commercial ELISA kits. To measure intracellular IL-17, single cell suspensions were obtained from MLN and restimulated for 5 h at 37°C in a tissue culture incubator with 10 ng/ml phorbol 12-myristate-13-acetate and 1 μg/ml ionomycin (Sigma) in the presence of 5 μg/ml of brefeldin A (BD Biosciences). Cells were then washed with PBS with 1% BSA and 2 mM EDTA, fixed with 3% formaldehyde in PBS, permeabilized with Permwash (BD Biosciences), and stained and analyzed by flow cytometry using a FACSCalibur and CellQuest software (BD Biosciences).

### Immunoblotting

For LC3B, inflammasome components and caspase-1 p10 detection, 1 x 10^6^ BMDCs were infected with STm or treated with alum in suspension, harvested by centrifugation at 200 x g at the indicated time points, and lysed in Laemmli sample buffer with 2-mercaptoethanol. Samples were then fractionated by SDS-PAGE on 15% polyacrylamide gels, transferred to PVDF membranes (Immobilon-FL, Millipore) and analyzed using Alexa Fluor 680 or 790-conjugated secondary antibodies (Jackson ImmunoResearch,) and Odyssey imaging system (LI-COR, Lincoln, NE). For IL-1β and caspase-1 p20 detection, samples were fractionated on 12% polyacrylamide gels and membranes were probed with horseradish peroxidase-conjugated secondary antibodies (Jackson ImmunoResearch) and enhanced chemiluminescence (GE Healthcare, Pittsburgh, PA). Densitometric analyses of band intensity was performed using NIH Image J software, normalizing to control protein levels [91].

### Cytokine secretion after TLR4 stimulation

BMDCs or BMMΦs were incubated with LPS-coated beads for 3 hr as described (Mantegazza 2012). Cytokine concentration in culture supernatants was measured by ELISA (ELISA Ready-SET-Go! Mouse TNFα, mouse interleukin-6, and mouse interleukins-12 and -23 [total p40], eBioscience).

### Intracellular bacteria replication assay

BMDCs (1x10^6^/ well) were seeded in 24-well plates in 1 ml RPMI medium in triplicate. Cells were infected at MOI 10:1 with flagellin-expressing STm. Gentamycin (100 μg/ml) was added 1 h after infection, and cells were collected 2 h after infection. Cells were then lysed in PBS/ 0.5% Triton X-100, and serial dilutions in PBS were plated on streptomycin-selective LB plates to determine bacterial load (CFU per ml).

### Cell death assay

Cytotoxicity was detected using the LDH Cytotoxicity Detection Kit (Clontech Laboratories, Inc., Mountain View, CA). 100,000/ well BMDCs were seeded into 96-well plates in RPMI medium in triplicates. Cells were infected at MOI 10:1 with flagellin-expressing STm. Gentamycin (100 μg/ml) was added 1 h after infection, and supernatants were harvested 2 h after infection. LDH release was quantified according to the manufacturer’s instructions. Cytotoxicity was normalized to cell samples treated with 1% (v/v) Triton X-100, and LDH release from uninfected cells was used for background subtraction.

### Live cell imaging

BMDCs or BMMΦs expressing ASC-GFP and/or mcherry-LC3b were seeded on poly-L-lysine–coated glass-bottom 35-mm culture dishes (MatTek, Ashland, MA) on day 6 of culture. On day 7, cells were infected with STm or mcherry-STm as described above under **Infections**. Cells were washed with RPMI and visualized by spinning-disk confocal microscopy using an Olympus inverted microscope equipped with an environmental chamber at 37°C and 5% CO2 and a Hamamatsu ImagEM EMCCD camera at the University of Pennsylvania’s Confocal Microscopy Core. Images or videos were obtained with MetaMorph software (Molecular Devices, Sunnyvale, CA) and analyzed using ImageJ (National Institutes of Health).

### Immunofluorescence microscopy and flow cytometry

ASC-GFP expressing BMDCs were infected with *Salmonella* for the indicated time points, fixed with 3% formaldehyde in PBS, permeabilized with Permwash (BD Biosciences, San Jose, CA), and labeled with primary antibodies and Alexa Fluor-conjugated secondary antibodies (Jackson ImmunoResearch). Cells were analyzed by fluorescence microscopy using a Zeiss LSM 710 laser-scanning confocal microscope and Zeiss ZEN 2012 software or using a Leica DMI6000 B inverted microscope, a Hamamatsu ORCA-flash4.0 camera and Leica Microsystems Application Suite X software. Images were analyzed using ImageJ. LC3b or p62 puncta were counted using Cell count plugin [80]. ASC speck distance from nuclei was quantified using Analyze/Measure tools as detailed in Image J tutorial (https://imagej.nih.gov/ij/docs/pdfs/ImageJ.pdf). The number of total p62 puncta was normalized to cell area.

To assess ASC speck accessibility to antibody detection by flow cytometry, BMDCs were infected with *Salmonella* for the indicated time points, washed with pre-warmed KHM buffer (110 mM CH_3_CO_2_K, 20 mM HEPES, 2 mM MgCl_2_, pH 7.3), permeablized with digitonin (50 μg/ml) or 0.1% saponin in KHM buffer for 1 min at room temperature and immediately washed with KHM buffer. Cells were then incubated sequentially with primary and secondary antibodies in 3% BSA in KHM buffer alone or supplemented with 0.1% saponin for 15 min at 37°C [48]. Cells were finally washed in PBS with 0.5% BSA and 2 mM EDTA and analyzed by flow cytometry using a FACSCalibur and CellQuest software (BD Biosciences).

### Statistical analyses

Statistical significance for experimental samples relative to untreated or uninfected WT control (unless otherwise stated) was determined using the unpaired Student's t test and ANOVA.

## Supporting information

S1 Fig DC numbers and phenotype in tissues of WT and AP-3^-/-^ mice are similar (related to Fig 3). Phenotypic characterization of MLN, small intestine lamina propria and splenic DCs isolated from WT and AP-3^-/-^ (pe) mice. (**A, B)** Cell suspensions from MLN (upper panels), small intestine lamina propria (middle panels), and B and T cell-depleted spleen (lower panels) were analyzed by flow cytometry. **A.** Representative dot-plots showing percentage of CD11c^+^ (red dots) and CD103^+^ populations in MLN and lamina propria, and CD11c^hi^ and MHC-II^+^ populations in spleen (right), compared to unstained negative controls (left). **B.** Data from three independent experiments presented as mean ± SD. No significant differences were detected. Average total cell numbers: 10x10^6^ (MLN), 30x10^6^ (small intestine), and 150x10^6^ (spleen; 5x10^6^ after depletion). (**C, D)**. Gated CD11c^+^ cells from MLN (upper panels), small intestine (middle panels) and spleen (bottom panels) from WT and pe mice. Cells were left untreated or infected with non-flagellin expressing STm for 6 h to induce cell maturation and prevent cell death. **C**. Representative dot-plots showing percentage of CCR7^+^ CD103^+^ (top left panels) and CCR7^+^ CD11c^+^ (bottom left panels), or CD86^+^ CD40^+^ (right panels) cells. **D**. Data from three independent experiments presented as mean ± SD. No significant differences were detected between WT and AP-3^-/-^ cells.
(TIF)

S2 Fig Serum IL-18 correlates with bacterial load in pearl mice 5 days after sublethal *Salmonella* Typhimurium infection (related to Fig 3). WT and pearl (pe) mice were infected orally with 10^8^ STm (+ STm) or treated with PBS as a control (naïve), and analyzed five days after infection. **A**. Blood was collected by cardiac puncture, and serum was isolated and assayed for IL-18 by ELISA. Data are pooled from three independent experiments and expressed as pg IL-18/ ml serum. **B-D**. Supernatants from homogenized and pelleted MLN were assayed for IL-18 (**B**), IL-1β (**C**) and IL-17 (**D**) in one experiment. Dotted lines, background signal threshold from uninfected mice; solid lines, mean value. *p<0.05; n.s., not significant.
(TIF)

S3 Fig Inflammasome activation is impaired in AP-3-deficient dendritic cells but not macrophages (related to Fig 4). **A.** BMDCs (DCs) or BMMΦs (MΦs) from WT and pearl (pe) mice were infected with STm at a MOI of 10:1. Cell supernatants collected after 4 h were assayed for IL-1β by ELISA. (**B-D**) WT and pearl (pe) mice were infected intranasally with 5 × 10^6^ *L*. *pneumophila* Δ*flaA (+ LPm)* or received PBS as control (naïve). **B.** Lung homogenates were plated to measure bacterial load, expressed as CFU/ g of lung. (**C, D**). Bronchoalveolar lavage (BAL) was assayed for TNFα (**D**) or IL-18 (**E**) by ELISA. (**B-D**). Dotted lines, background (threshold values from uninfected mice); solid lines, geometric mean (**B**), or arithmetic mean (**C**, **D**) of values above background. ***p<0.001; n.s., not significant.
(TIF)

S4 Fig AP-3 does not affect phagosomal TLR signaling in MΦs (related to Fig 4). BMDCs (**A**, **C**, **E**) or BMMΦs (**B**, **D**, **F**) were incubated for 3 h with LPS-coated or uncoated latex beads, and TNFα (**A**, **B**), IL-6 (**C**, **D**) and IL-12p40 (**E**, **F**) were measured in cell supernatants by ELISA. Data from three independent experiments are normalized to LPS-coated bead-treated WT cells as 100% and represented as mean ± SD. ***p<0.001.
(TIF)

S5 Fig AP-3 is required for perinuclear inflammasome positioning in response to multiple stimuli in DCs and MΦs. (related to Fig 5). WT and pearl (pe) BMDCs (**A**-**C**) or BMMΦs (**D**, **E**) expressing ASC-GFP were analyzed by fluorescence microscopy. **A**. Representative images of uninfected BMDCs. **B**. BMDCs were infected with mCherry-STm and cells were analyzed at the indicated times after infection. ASC specks were quantified in 20 cells per cell type in each of three independent experiments. Data are presented as mean ± SD. No significant differences between WT and pearl cells were observed. **C-E**. BMDCs (**C**) or BMMΦs (**D, E**) were primed with LPS for 3 h and stimulated with ATP for 30 min (**C** *left*) or alum for 5 h (**C, D** *right*), or infected with STm for 1 h (**D** left). (**C-D**) Representative images showing ASC speck location (green) relative to whole cells visualized by DIC in WT and pearl cells. (**E**) Quantification of perinuclear (within a radius of one μμ from the nucleus) and non-perinuclear ASC specks in 15 BMMΦs per cell type in each of three independent experiments. N, nucleus. Scale bar: 10 μm. ***, p<0.001.
(TIF)

S6 Fig AP-3 limits autophagy induction in DCs but not in MΦs after inflammasome activation with alum but not with ATP (related to Fig 5). **A-D, F-I.** WT and pearl (pe) BMDCs (**A-D**) or BMMΦs (**F-I**) were primed with LPS for 3 h and stimulated with alum (**A**, **B, H, I**) or ATP (**C**, **D**), or infected with STm (**F, G**). Endogenous LC3 and β-actin or γ-tubulin as a loading control were detected by immunoblotting cell lysates at the indicated time points. **A, C, F, H.** Shown are representative blots, with the actin, tubulin and LC3-I and LC3-II bands highlighted. Positions of nearby molecular weight markers (MW) are shown to the left. **B, D, G, I**. Quantification of LC3-II band intensities from three independent experiments, expressed as fold increase relative to unstimulated cells and normalized to LC3-I and either β-actin or γ-tubulin levels. **E.** WT and pearl (pe) BMDCs expressing ASC-GFP alone or with mCherry-LC3 were infected with STm and analyzed by live imaging (for LC3) or immunofluorescence microscopy on fixed cells (for p62) 1 h later. Representative images showing ASC speck (green) and either LC3 puncta (red, *left panels*) or endogenous p62 puncta (red) relative to the nucleus labeled with DAPI (*right panels*) in infected cells. DIC images are shown to emphasize nuclear position. Data represent mean ± SD. Scale bars: 10 μm. *p<0.05; **p<0.01.
(TIF)

S7 Fig Impaired production of IL-1β by AP-3-deficient cells after STm infection or alum stimulation, but not of TNFα or pro-IL-1β after priming, requires autophagy induction (related to Fig 6). (**A, B)**. WT and pearl (pe) BMDCs expressing ASC-GFP were infected with flagellin-expressing STm (to stimulate NLRC4 inflammasome) for 1 h, then treated either for 1 min with 50 μg/ml digitonin or throughout labeling with 0.1% saponin. **A.** Cells were then stained with a rabbit antibody to the cytoplasmic tail of the ER transmembrane protein, calnexin, and APC-conjugated anti rabbit antibody, or with a mouse antibody to the ER luminal enzyme, protein disulfide isomerase (PDI), and APC-conjugated anti mouse antibody. Cells were analyzed by flow cytometry; representative histogram plots are shown. *Black lines*, WT; *blue lines*, pe. *Dotted lines*, secondary antibody alone. **B**. WT (top) or pearl (pe, bottom) BMDCs expressing ASC-GFP were fixed and stained with AF594-labeled anti-GFP antibody and analyzed by IFM. Shown are individual and merged fluorescence images and corresponding DIC image at right. (**C-J**) WT and pearl (pe) BMDCs that were non-transduced (-) or transduced with lentiviruses encoding non-target (ctrl) or either of two ATG7-specific or ATG5- or LC3b- specific shRNAs were untreated (**C**, **I**, **J**) or infected with flagellin-expressing STm (MOI 10:1) (**D-F**), stimulated with LPS-beads (**G**, **H**), or primed for 3 h with LPS and stimulated with alum (**K-M**). (**C, I, J**). Representative immunoblots of cell lysates for ATG7, ATG5, LC3b or tubulin as a loading control. **D**. After 2h, cells were lysed with 0.5% Triton X-100 in PBS and serial dilutions were plated in streptomycin containing agar plates to assess colony formation. Data from 3 independent experiments are shown. **E.** After 2 h, cells were pelleted and LDH release into the supernatant was measured. Percent of LDH release was normalized to release from uninfected cells by 1% Triton X-100 treatment (Triton), and LDH release from uninfected cells was subtracted as background. Data from 3 independent experiments are shown **F.** Cell supernatants collected 2 h after Stm infection were assayed for TNFα by ELISA. (**G, H**) Cells were stimulated with LPS-beads for 3 h. **G**. Representative immunoblot for pro-IL-1β induction after priming and for tubulin as a loading control. **H**. Quantification of band intensities for pro-IL-1β, presented as mean ± SD fold induction in stimulated cells relative to unstimulated cells from three independent experiments (**K-M**). Cell supernatants collected 4 h after alum stimulation were assayed for IL-1β by ELISA in triplicate. **K**. Shown is a representative of 3 independent experiments. **L**. Data from 3 independent experiments were normalized to IL-1β values from cells treated with non-target shRNA and presented as fold induction. **M**. IL-1β values for pearl BMDCs treated with non-target, ATG5 or LC3b shRNAs from 3 independent experiments are shown as percent of values for WT DCs treated with the same shRNAs. Data in **D-F**, **H, K-M** are presented as mean ± SD. *p<0.05; ***p<0.001.
(TIF)

S1 Table AP-3 is required for optimal transcriptional activation of proinflammatory cytokines and some NLRs after particulate LPS priming (related to Fig 1; see associated Excel file). WT (columns A-G) and pearl (columns H-N) BMDCs were untreated or stimulated with LPS beads (beads) or soluble LPS (soluble) for 2h. cDNA generated from isolated RNA was measured by RT-PCR and analysed as described. Relative levels of different cytokines and chemokines (columns P-AC) were calculated as mRNA fold-induction compared to unstimulated cells.
(XLSX)

## References

[1] Blander JM, Sander LE. Beyond pattern recognition: five immune checkpoints for scaling the microbial threat. Nat Rev Immunol. 2012;12(3):215–25. doi: 10.1038/nri3167 . 22362354 10.1038/nri3167

[2] Vance RE, Isberg RR, Portnoy DA. Patterns of pathogenesis: discrimination of pathogenic and nonpathogenic microbes by the innate immune system. Cell Host Microbe. 2009;6(1):10–21. doi: 10.1016/j.chom.2009.06.007 . 19616762 10.1016/j.chom.2009.06.007 PMC2777727

[3] Kawai T, Akira S. The role of pattern-recognition receptors in innate immunity: update on Toll-like receptors. Nat Immunol. 2010;11(5):373–84. doi: 10.1038/ni.1863 . 20404851 10.1038/ni.1863

[4] Hornung V, Latz E. Critical functions of priming and lysosomal damage for NLRP3 activation. Eur J Immunol. 2010;40(3):620–3. doi: 10.1002/eji.200940185 ; PubMed Central PMCID: PMC3893565. 20201015 10.1002/eji.200940185 PMC3893565

[5] Franchi L, Munoz-Planillo R, Nunez G. Sensing and reacting to microbes through the inflammasomes. Nat Immunol. 2012;13(4):325–32. doi: 10.1038/ni.2231 . 22430785 10.1038/ni.2231 PMC3449002

[6] Schroder K, Tschopp J. The inflammasomes. Cell. 2010;140(6):821–32. doi: 10.1016/j.cell.2010.01.040 . 20303873 10.1016/j.cell.2010.01.040

[7] Horvath GL, Schrum JE, De Nardo CM, Latz E. Intracellular sensing of microbes and danger signals by the inflammasomes. Immunol Rev. 2011;243(1):119–35. doi: 10.1111/j.1600-065X.2011.01050.x ; PubMed Central PMCID: PMC3893570. 21884172 10.1111/j.1600-065X.2011.01050.x PMC3893570

[8] Latz E, Xiao TS, Stutz A. Activation and regulation of the inflammasomes. Nat Rev Immunol. 2013;13(6):397–411. doi: 10.1038/nri3452 ; PubMed Central PMCID: PMC3807999. 23702978 10.1038/nri3452 PMC3807999

[9] Puren AJ, Fantuzzi G, Dinarello CA. Gene expression, synthesis, and secretion of interleukin 18 and interleukin 1beta are differentially regulated in human blood mononuclear cells and mouse spleen cells. Proceedings of the National Academy of Sciences of the United States of America. 1999;96(5):2256–61. ; PubMed Central PMCID: PMC26770. 10051628 10.1073/pnas.96.5.2256 PMC26770

[10] Mariathasan S, Monack DM. Inflammasome adaptors and sensors: intracellular regulators of infection and inflammation. Nat Rev Immunol. 2007;7(1):31–40. doi: 10.1038/nri1997 . 17186029 10.1038/nri1997

[11] Moretti J, Blander JM. Insights into phagocytosis-coupled activation of pattern recognition receptors and inflammasomes. Curr Opin Immunol. 2014;26:100–10. doi: 10.1016/j.coi.2013.11.003 ; PubMed Central PMCID: PMC3932007. 24556406 10.1016/j.coi.2013.11.003 PMC3932007

[12] Rathinam VA, Vanaja SK, Fitzgerald KA. Regulation of inflammasome signaling. Nature immunology. 2012;13(4):333–42. doi: 10.1038/ni.2237 ; PubMed Central PMCID: PMC3523703. 22430786 10.1038/ni.2237 PMC3523703

[13] Saitoh T, Fujita N, Jang MH, Uematsu S, Yang BG, Satoh T, et al Loss of the autophagy protein Atg16L1 enhances endotoxin-induced IL-1beta production. Nature. 2008;456(7219):264–8. doi: 10.1038/nature07383 . 18849965 10.1038/nature07383

[14] Shi CS, Shenderov K, Huang NN, Kabat J, Abu-Asab M, Fitzgerald KA, et al Activation of autophagy by inflammatory signals limits IL-1beta production by targeting ubiquitinated inflammasomes for destruction. Nature immunology. 2012;13(3):255–63. doi: 10.1038/ni.2215 ; PubMed Central PMCID: PMC4116819. 22286270 10.1038/ni.2215 PMC4116819

[15] Tattoli I, Sorbara MT, Vuckovic D, Ling A, Soares F, Carneiro LA, et al Amino acid starvation induced by invasive bacterial pathogens triggers an innate host defense program. Cell Host Microbe. 2012;11(6):563–75. doi: 10.1016/j.chom.2012.04.012 . 22704617 10.1016/j.chom.2012.04.012

[16] Thurston TL, Boyle KB, Allen M, Ravenhill BJ, Karpiyevich M, Bloor S, et al Recruitment of TBK1 to cytosol-invading Salmonella induces WIPI2-dependent antibacterial autophagy. EMBO J. 2016;35(16):1779–92. doi: 10.15252/embj.201694491 . 27370208 10.15252/embj.201694491 PMC5010046

[17] Huang J, Brumell JH. Bacteria-autophagy interplay: a battle for survival. Nat Rev Microbiol. 2014;12(2):101–14. doi: 10.1038/nrmicro3160 . 24384599 10.1038/nrmicro3160 PMC7097477

[18] Mantegazza AR, Guttentag SH, El-Benna J, Sasai M, Iwasaki A, Shen H, et al Adaptor protein-3 in dendritic cells facilitates phagosomal toll-like receptor signaling and antigen presentation to CD4(+) T cells. Immunity. 2012;36(5):782–94. doi: 10.1016/j.immuni.2012.02.018 . 22560444 10.1016/j.immuni.2012.02.018 PMC3361531

[19] Huizing M, Helip-Wooley A, Westbroek W, Gunay-Aygun M, Gahl WA. Disorders of lysosome-related organelle biogenesis: clinical and molecular genetics. Annu Rev Genomics Hum Genet. 2008;9:359–86. doi: 10.1146/annurev.genom.9.081307.164303 ; PubMed Central PMCID: PMCPMC2755194. 18544035 10.1146/annurev.genom.9.081307.164303 PMC2755194

[20] Wei A-H, Li W. Hermansky-Pudlak syndrome: Pigmentary and non-pigmentary defects and their pathogenesis. Pigment Cell Melanoma Res. 2013;26(2):176–92. doi: 10.1111/pcmr.12051 . 23171219 10.1111/pcmr.12051

[21] Lin K-M, Hu W, Troutman TD, Jennings M,., Brewer T, Li X, et al IRAK-1 bypasses priming and directly links TLRs to rapid NLRP3 inflammasome activation. Proc Natl Acad Sci USA. 2014;111(2):775–80. doi: 10.1073/pnas.1320294111 ; PubMed Central PMCID: PMCPMC3896167. 24379360 10.1073/pnas.1320294111 PMC3896167

[22] Petrilli V, Papin S, Dostert C, Mayor A, Martinon F, Tschopp J. Activation of the NALP3 inflammasome is triggered by low intracellular potassium concentration. Cell Death Differ. 2007;14(9):1583–9. doi: 10.1038/sj.cdd.4402195 . 17599094 10.1038/sj.cdd.4402195

[23] Wynosky-Dolfi MA, Snyder AG, Philip NH, Doonan PJ, Poffenberger MC, Avizonis D, et al Oxidative metabolism enables Salmonella evasion of the NLRP3 inflammasome. J Exp Med. 2014;211(4):653–68. doi: 10.1084/jem.20130627 ; PubMed Central PMCID: PMC3978275. 24638169 10.1084/jem.20130627 PMC3978275

[24] Lin KM, Hu W, Troutman TD, Jennings M, Brewer T, Li X, et al IRAK-1 bypasses priming and directly links TLRs to rapid NLRP3 inflammasome activation. Proceedings of the National Academy of Sciences of the United States of America. 2014;111(2):775–80. doi: 10.1073/pnas.1320294111 ; PubMed Central PMCID: PMCPMC3896167. 24379360 10.1073/pnas.1320294111 PMC3896167

[25] Stoll S, Jonuleit H, Schmitt E, Müller G, Yamauchi H, Kurimoto M, et al Production of functional IL-18 by different subtypes of murine and human dendritic cells (DC): DC-derived IL-18 enhances IL-12-dependent Th1 development. Eur J Immunol. 1998;28(10):3231–9. doi: 10.1002/(SICI)1521-4141(199810)28:10<3231::AID-IMMU3231>3.0.CO;2-Q . 9808192 10.1002/(SICI)1521-4141(199810)28:10<3231::AID-IMMU3231>3.0.CO;2-Q

[26] Gardella S, Andrei C, Costigliolo S, Poggi A, Zocchi MR, Rubartelli A. Interleukin-18 synthesis and secretion by dendritic cells are modulated by interaction with antigen-specific T cells. J Leukoc Biol. 1999;66(2):237–41. 10449160

[27] Lee SJ, McLachlan JB, Kurtz JR, Fan D, Winter SE, Baumler AJ, et al Temporal expression of bacterial proteins instructs host CD4 T cell expansion and Th17 development. PLoS pathogens. 2012;8(1):e1002499 doi: 10.1371/journal.ppat.1002499 ; PubMed Central PMCID: PMC3262010. 22275869 10.1371/journal.ppat.1002499 PMC3262010

[28] Tam MA, Rydstrom A, Sundquist M, Wick MJ. Early cellular responses to Salmonella infection: dendritic cells, monocytes, and more. Immunol Rev. 2008;225:140–62. doi: 10.1111/j.1600-065X.2008.00679.x . 18837781 10.1111/j.1600-065X.2008.00679.x

[29] Niedergang F, Sirard JC, Blanc CT, Kraehenbuhl JP. Entry and survival of Salmonella typhimurium in dendritic cells and presentation of recombinant antigens do not require macrophage-specific virulence factors. Proceedings of the National Academy of Sciences of the United States of America. 2000;97(26):14650–5. doi: 10.1073/pnas.97.26.14650 ; PubMed Central PMCID: PMC18973. 11121065 10.1073/pnas.97.26.14650 PMC18973

[30] McSorley SJ. Immunity to intestinal pathogens: lessons learned from Salmonella. Immunol Rev. 2014;260(1):168–82. doi: 10.1111/imr.12184 ; PubMed Central PMCID: PMC4066191. 24942689 10.1111/imr.12184 PMC4066191

[31] Sozzani S, Allavena P, D'Amico G, Luini W, Bianchi G, Kataura M, et al Differential regulation of chemokine receptors during dendritic cell maturation: a model for their trafficking properties. Journal of immunology. 1998;161(3):1083–6. . 9686565

[32] Jang MH, Sougawa N, Tanaka T, Hirata T, Hiroi T, Tohya K, et al CCR7 is critically important for migration of dendritic cells in intestinal lamina propria to mesenteric lymph nodes. J Immunol. 2006;176(2):803–10. . 16393963 10.4049/jimmunol.176.2.803

[33] Shaw MH, Kamada N, Kim YG, Núñez G. Microbiota-induced IL-1β, but not IL-6, is critical for the development of steady-state TH17 cells in the intestine. J Exp Med. 2012;209(2):251–8. doi: 10.1084/jem.20111703 ; PubMed Central PMCID: PMCPMC3280878. 22291094 10.1084/jem.20111703 PMC3280878

[34] Zanoni I, Tan Y, Di Gioia M, Broggi A, Ruan J, Shi J, et al An endogenous caspase-11 ligand elicits interleukin-1 release from living dendritic cells. Science. 2016;352(6290):1232–6. doi: 10.1126/science.aaf3036 . 27103670 10.1126/science.aaf3036 PMC5111085

[35] Copenhaver AM, Casson CN, Nguyen HT, Fung TC, Duda MM, Roy CR, et al Alveolar macrophages and neutrophils are the primary reservoirs for Legionella pneumophila and mediate cytosolic surveillance of type IV secretion. Infect Immun. 2014;82(10):4325–36. doi: 10.1128/IAI.01891-14 ; PubMed Central PMCID: PMC4187856. 25092908 10.1128/IAI.01891-14 PMC4187856

[36] Case CL, Kohler LJ, Lima JB, Strowig T, de Zoete MR, Flavell RA, et al Caspase-11 stimulates rapid flagellin-independent pyroptosis in response to Legionella pneumophila. Proceedings of the National Academy of Sciences of the United States of America. 2013;110(5):1851–6. doi: 10.1073/pnas.1211521110 ; PubMed Central PMCID: PMC3562791. 23307811 10.1073/pnas.1211521110 PMC3562791

[37] Casson CN, Copenhaver AM, Zwack EE, Nguyen HT, Strowig T, Javdan B, et al Caspase-11 activation in response to bacterial secretion systems that access the host cytosol. PLoS pathogens. 2013;9(6):e1003400 doi: 10.1371/journal.ppat.1003400 ; PubMed Central PMCID: PMC3675167. 23762026 10.1371/journal.ppat.1003400 PMC3675167

[38] Dell'Angelica EC. AP-3-dependent trafficking and disease: the first decade. Curr Opin Cell Biol. 2009;21(4):552–9. doi: 10.1016/j.ceb.2009.04.014 . 19497727 10.1016/j.ceb.2009.04.014

[39] Clark RH, Stinchcombe JC, Day A, Blott E, Booth S, Bossi G, et al Adaptor protein 3-dependent microtubule-mediated movement of lytic granules to the immunological synapse. Nature immunology. 2003;4(11):1111–20. doi: 10.1038/ni1000 . 14566336 10.1038/ni1000

[40] Ivan V, Martinez-Sanchez E, Sima LE, Oorschot V, Klumperman J, Petrescu SM, et al AP-3 and Rabip4' coordinately regulate spatial distribution of lysosomes. PLoS One. 2012;7(10):e48142 doi: 10.1371/journal.pone.0048142 ; PubMed Central PMCID: PMC3483219. 23144738 10.1371/journal.pone.0048142 PMC3483219

[41] Dick MS, Sborgi L, Ruhl S, Hiller S, Broz P. ASC filament formation serves as a signal amplification mechanism for inflammasomes. Nat Commun. 2016;7:11929 doi: 10.1038/ncomms11929 ; PubMed Central PMCID: PMC4917984. 27329339 10.1038/ncomms11929 PMC4917984

[42] Stutz A, Horvath GL, Monks BG, Latz E. ASC speck formation as a readout for inflammasome activation. Methods Mol Biol. 2013;1040:91–101. doi: 10.1007/978-1-62703-523-1_8 . 23852599 10.1007/978-1-62703-523-1_8

[43] Broz P, Monack DM. Measuring inflammasome activation in response to bacterial infection. Methods Mol Biol. 2013;1040:65–84. doi: 10.1007/978-1-62703-523-1_6 . 23852597 10.1007/978-1-62703-523-1_6

[44] Martin BN, Wang C, Willette-Brown J, Herjan T, Gulen MF, Zhou H, et al IKKalpha negatively regulates ASC-dependent inflammasome activation. Nat Commun. 2014;5:4977 doi: 10.1038/ncomms5977 ; PubMed Central PMCID: PMC4298287. 25266676 10.1038/ncomms5977 PMC4298287

[45] Jessop F, Hamilton RF, Rhoderick JF, Shaw PK, Holian A. Autophagy deficiency in macrophages enhances NLRP3 inflammasome activity and chronic lung disease following silica exposure. Toxicol Appl Pharmacol. 2016;309:101–10. doi: 10.1016/j.taap.2016.08.029 ; PubMed Central PMCID: PMCPMC5054752. 27594529 10.1016/j.taap.2016.08.029 PMC5054752

[46] Nurmi K, Kareinen I, Virkanen J, Rajamäki K, Kouri VP, Vaali K, et al Hemin and Cobalt Protoporphyrin Inhibit NLRP3 Inflammasome Activation by Enhancing Autophagy: A Novel Mechanism of Inflammasome Regulation. J Innate Immun. 2017;9(1):65–82. doi: 10.1159/000448894 . 27655219 10.1159/000448894 PMC6738905

[47] Nakahira K, Haspel JA, Rathinam VA, Lee SJ, Dolinay T, Lam HC, et al Autophagy proteins regulate innate immune responses by inhibiting the release of mitochondrial DNA mediated by the NALP3 inflammasome. Nat Immunol. 2011;12(3):222–30. doi: 10.1038/ni.1980 ; PubMed Central PMCID: PMCPMC3079381. 21151103 10.1038/ni.1980 PMC3079381

[48] Meunier E, Broz P. Quantification of cytosolic vs. vacuolar Salmonella in primary macrophages by differential permeabilization. J VisExp. 2015;(101):e52960 doi: 10.3791/52960 . 26274778 10.3791/52960 PMC4545148

[49] Miao EA, Leaf IA, Treuting PM, Mao DP, Dors M, Sarkar A, et al Caspase-1-induced pyroptosis is an innate immune effector mechanism against intracellular bacteria. Nature immunology. 2010;11(12):1136–42. doi: 10.1038/ni.1960 ; PubMed Central PMCID: PMC3058225. 21057511 10.1038/ni.1960 PMC3058225

[50] Franchi L, Kamada N, Nakamura Y, Burberry A, Kuffa P, Suzuki S, et al NLRC4-driven production of IL-1beta discriminates between pathogenic and commensal bacteria and promotes host intestinal defense. Nature immunology. 2012;13(5):449–56. doi: 10.1038/ni.2263 ; PubMed Central PMCID: PMC3361590. 22484733 10.1038/ni.2263 PMC3361590

[51] Saitoh T, Akira S. Regulation of inflammasomes by autophagy. J Allergy Clin Immunol. 2016;138(1):28–36. doi: 10.1016/j.jaci.2016.05.009 . 27373323 10.1016/j.jaci.2016.05.009

[52] Harris J, Hartman M, Roche C, Zeng SG, O’Shea A, Sharp FA, et al Autophagy controls IL-1β secretion by targeting Pro-IL-1 β for degradation. J Biol Chem. 2011;286(11):9587–97. doi: 10.1074/jbc.M110.202911 ; PubMed Central PMCID: PMCPMC3058966. 21228274 10.1074/jbc.M110.202911 PMC3058966

[53] Bauernfeind FG, Horvath G, Stutz A, Alnemri ES, MacDonald K, Speert D, et al Cutting edge: NF-kappaB activating pattern recognition and cytokine receptors license NLRP3 inflammasome activation by regulating NLRP3 expression. Journal of immunology. 2009;183(2):787–91. doi: 10.4049/jimmunol.0901363 ; PubMed Central PMCID: PMC2824855. 19570822 10.4049/jimmunol.0901363 PMC2824855

[54] Fontana S, Parolini S, Vermi W, Booth S, Gallo F, Donini M, et al Innate immunity defects in Hermansky-Pudlak type 2 syndrome. Blood. 2006;107(12):4857–64. doi: 10.1182/blood-2005-11-4398 . 16507770 10.1182/blood-2005-11-4398

[55] Broz P, Newton K, Lamkanfi M, Mariathasan S, Dixit VM, Monack DM. Redundant roles for inflammasome receptors NLRP3 and NLRC4 in host defense against Salmonella. J Exp Med. 2010;207(8):1745–55. doi: 10.1084/jem.20100257 . 20603313 10.1084/jem.20100257 PMC2916133

[56] Ravindran R, Loebbermann J, Nakaya HI, Khan N, Ma H, Gama L, et al The amino acid sensor GCN2 controls gut inflammation by inhibiting inflammasome activation. Nature. 2016;531(7595):523–7. doi: 10.1038/nature17186 ; PubMed Central PMCID: PMC PMC4854628. 26982722 10.1038/nature17186 PMC4854628

[57] Kimura T, Jain A, Choi SW, Mandell MA, Schroder K, Johansen T, et al TRIM-mediated precision autophagy targets cytoplasmic regulators of innate immunity. J Cell Biol. 2015;210(6):973–89. doi: 10.1083/jcb.201503023 ; PubMed Central PMCID: PMC4576868. 26347139 10.1083/jcb.201503023 PMC4576868

[58] Zhong Z, Umemura A, Sanchez-Lopez E, Liang S, Shalapour S, Wong J, et al NF-κB restricts inflammasome activation via elimination of damaged mitochondria. Cell. 2016;164(5):896–910. doi: 10.1016/j.cell.2015.12.057 ; PubMed Central PMCID: PMCPMC4769378. 26919428 10.1016/j.cell.2015.12.057 PMC4769378

[59] Jin M, Klionsky DJ. Regulation of autophagy: modulation of the size and number of autophagosomes. FEBS Lett. 2014;588(15):2457–63. doi: 10.1016/j.febslet.2014.06.015 ; PubMed Central PMCID: PMCPMC4118767. 24928445 10.1016/j.febslet.2014.06.015 PMC4118767

[60] Cai X, Chen J, Xu H, Liu S, Jiang QX, Halfmann R, et al Prion-like polymerization underlies signal transduction in antiviral immune defense and inflammasome activation. Cell. 2014;156(6):1207–22. doi: 10.1016/j.cell.2014.01.063 ; PubMed Central PMCID: PMCPMC4034535. 24630723 10.1016/j.cell.2014.01.063 PMC4034535

[61] Mollenhauer HH, Morré DJ. Structural compartmentation of the cytosol: zones of exclusion, zones of adhesion, cytoskeletal and intercisternal elements. Subcell Biochem. 1978;5:327–59. . 97812 10.1007/978-1-4615-7942-7_7

[62] Sitaram A, Dennis MK, Chaudhuri R, De Jesus-Rojas W, Tenza D, Setty SR, et al Differential recognition of a dileucine-based sorting signal by AP-1 and AP-3 reveals a requirement for both BLOC-1 and AP-3 in delivery of OCA2 to melanosomes. Mol Biol Cell. 2012;23(16):3178–92. doi: 10.1091/mbc.E11-06-0509 ; PubMed Central PMCID: PMC3418312. 22718909 10.1091/mbc.E11-06-0509 PMC3418312

[63] Salazar G, Love R, Styers ML, Werner E, Peden A, Rodriguez S, et al AP-3-dependent mechanisms control the targeting of a chloride channel (ClC-3) in neuronal and non-neuronal cells. J Biol Chem. 2004;279(24):25430–9. PubMed Central PMCID: PMC15073168. doi: 10.1074/jbc.M402331200 15073168 10.1074/jbc.M402331200

[64] Chantranupong L, Wolfson RL, Sabatini DM. Nutrient-sensing mechanisms across evolution. Cell. 2015;161(1):67–83. doi: 10.1016/j.cell.2015.02.041 ; PubMed Central PMCID: PMCPMC4384161. 25815986 10.1016/j.cell.2015.02.041 PMC4384161

[65] Savina A, Jancic C, Hugues S, Guermonprez P, Vargas P, Moura IC, et al NOX2 controls phagosomal pH to regulate antigen processing during crosspresentation by dendritic cells. Cell. 2006;126(1):205–18. doi: 10.1016/j.cell.2006.05.035 . 16839887 10.1016/j.cell.2006.05.035

[66] Savina A, Amigorena S. Phagocytosis and antigen presentation in dendritic cells. Immunol Rev. 2007;219:143–56. doi: 10.1111/j.1600-065X.2007.00552.x . 17850487 10.1111/j.1600-065X.2007.00552.x

[67] Flannagan RS, Jaumouillé V, Grinstein S. The cell biology of phagocytosis. Annu Rev Pathol: Mechanisms of Disease. 2012;7:61–98. doi: 10.1146/annurev-pathol-011811-132445 . 21910624 10.1146/annurev-pathol-011811-132445

[68] Mantegazza AR, Zajac AL, Twelvetrees A, Holzbaur EL, Amigorena S, Marks MS. TLR-dependent phagosome tubulation in dendritic cells promotes phagosome cross-talk to optimize MHC-II antigen presentation. Proceedings of the National Academy of Sciences of the United States of America. 2014;111(43):15508–13. doi: 10.1073/pnas.1412998111 ; PubMed Central PMCID: PMC4217451. 25313083 10.1073/pnas.1412998111 PMC4217451

[69] Lee HK, Mattei LM, Steinberg BE, Alberts P, Lee YH, Chervonsky A, et al In vivo requirement for Atg5 in antigen presentation by dendritic cells. Immunity. 2010;32(2):227–39. PubMed Central PMCID: PMCPMC2996467. doi: 10.1016/j.immuni.2009.12.006 20171125 10.1016/j.immuni.2009.12.006 PMC2996467

[70] Sanjuan MA, Dillon CP, Tait SW, Moshiach S, Dorsey F, Connell S, et al Toll-like receptor signalling in macrophages links the autophagy pathway to phagocytosis. Nature. 2007;450(7173):1253–7. doi: 10.1038/nature06421 . 18097414 10.1038/nature06421

[71] Cemma M, Grinstein S, Brumell JH. Autophagy proteins are not universally required for phagosome maturation. Autophagy. 2016;12(9):1440–6. doi: 10.1080/15548627.2016.1191724 . 27310610 10.1080/15548627.2016.1191724 PMC5082775

[72] Dell'Angelica EC, Shotelersuk V, Aguilar RC, Gahl WA, Bonifacino JS. Altered trafficking of lysosomal proteins in Hermansky-Pudlak syndrome due to mutations in the b3A subunit of the AP-3 adaptor. Mol Cell. 1999;3(1):11–21. . 10024875 10.1016/s1097-2765(00)80170-7

[73] Feng L, Seymour AB, Jiang S, To A, Peden AA, Novak EK, et al The b3A subunit gene (Ap3b1) of the AP-3 adaptor complex is altered in the mouse hypopigmentation mutant pearl, a model for Hermansky-Pudlak syndrome and night blindness. Hum Mol Genet. 1999;8(2):323–30. . 9931340 10.1093/hmg/8.2.323

[74] Meng G, Zhang F, Fuss I, Kitani A, Strober W. A mutation in the Nlrp3 gene causing inflammasome hyperactivation potentiates Th17 cell-dominant immune responses. Immunity. 2009;30(6):860–74. doi: 10.1016/j.immuni.2009.04.012 . 19501001 10.1016/j.immuni.2009.04.012 PMC2764254

[75] Aujla SJ, Chan YR, Zheng M, Fei M, Askew DJ, Pociask DA, et al IL-22 mediates mucosal host defense against Gram-negative bacterial pneumonia. Nat Med. 2008;14(3):275–81. doi: 10.1038/nm1710 ; PubMed Central PMCID: PMC2901867. 18264110 10.1038/nm1710 PMC2901867

[76] Ivanov II, Atarashi K, Manel N, Brodie EL, Shima T, Karaoz U, et al Induction of intestinal Th17 cells by segmented filamentous bacteria. Cell. 2009;139(3):485–98. doi: 10.1016/j.cell.2009.09.033 ; PubMed Central PMCID: PMC2796826. 19836068 10.1016/j.cell.2009.09.033 PMC2796826

[77] Wenham M, Grieve S, Cummins M, Jones ML, Booth S, Kilner R, et al Two patients with Hermansky Pudlak syndrome type 2 and novel mutations in AP3B1. Haematologica. 2010;95(2):333–7. doi: 10.3324/haematol.2009.012286 ; PubMed Central PMCID: PMCPMC2817039. 19679886 10.3324/haematol.2009.012286 PMC2817039

[78] Jessen B, Bode SF, Ammann S, Chakravorty S, Davies G, Diestelhorst J, et al The risk of hemophagocytic lymphohistiocytosis in Hermansky-Pudlak syndrome type 2. Blood. 2013;121(15):2943–51. doi: 10.1182/blood-2012-10-463166 ; PubMed Central PMCID: PMCPMC3624940. 23403622 10.1182/blood-2012-10-463166 PMC3624940

[79] Smith E, Zarbock A, Stark MA, Burcin TL, Bruce AC, Foley P, et al IL-23 is required for neutrophil homeostasis in normal and neutrophilic mice. J Immunol. 2007;179(12):8274–9. . 18056371 10.4049/jimmunol.179.12.8274

[80] Mantegazza AR, Marks MS. Visualizing toll-like receptor-dependent phagosomal dynamics in murine dendritic cells using live cell microscopy. Methods Mol Biol. 2015;1270:191–203. doi: 10.1007/978-1-4939-2309-0_15 ; PubMed Central PMCID: PMC4356480. 25702119 10.1007/978-1-4939-2309-0_15 PMC4356480

[81] Gedde MM, Higgins DE, Tilney LG, Portnoy DA. Role of listeriolysin O in cell-to-cell spread of Listeria monocytogenes. Infect Immun. 2000;68(2):999–1003. . 10639481 10.1128/iai.68.2.999-1003.2000 PMC97240

[82] Glomski IJ, Gedde MM, Tsang AW, Swanson JA, Portnoy DA. The Listeria monocytogenes hemolysin has an acidic pH optimum to compartmentalize activity and prevent damage to infected host cells. J Cell Biol. 2002;156(6):1029–38. doi: 10.1083/jcb.200201081 . 11901168 10.1083/jcb.200201081 PMC2173464

[83] Winzler C, Rovere P, Rescigno M, Granucci F, Penna G, Adorini L, et al Maturation stages of mouse dendritic cells in growth factor-dependent long-term cultures. J Exp Med. 1997;185(2):317–28. . 9016880 10.1084/jem.185.2.317 PMC2196118

[84] Savina A, Peres A, Cebrian I, Carmo N, Moita C, Hacohen N, et al The small GTPase Rac2 controls phagosomal alkalinization and antigen crosspresentation selectively in CD8(+) dendritic cells. Immunity. 2009;30(4):544–55. doi: 10.1016/j.immuni.2009.01.013 . 19328020 10.1016/j.immuni.2009.01.013

[85] Geem D, Medina-Contreras O, Kim W, Huang CS, Denning TL. Isolation and characterization of dendritic cells and macrophages from the mouse intestine. Journal of visualized experiments: JoVE. 2012;(63):e4040 doi: 10.3791/4040 ; PubMed Central PMCID: PMC3466926. 22644046 10.3791/4040 PMC3466926

[86] Morita S, Kojima T, Kitamura T. Plat-E: an efficient and stable system for transient packaging of retroviruses. Gene Ther. 2000;7(12):1063–6. doi: 10.1038/sj.gt.3301206 . 10871756 10.1038/sj.gt.3301206

[87] Stewart SA, Dykxhoorn DM, Palliser D, Mizuno H, Yu EY, An DS, et al Lentivirus-delivered stable gene silencing by RNAi in primary cells. RNA 2003; 9(4): 493–501. PMCID: 1370415 12649500 10.1261/rna.2192803 PMC1370415

[88] Marks MS, Roche PA, van Donselaar E, Woodruff L, Peters PJ, Bonifacino JS. A lysosomal targeting signal in the cytoplasmic tail of the b chain directs HLA-DM to the MHC class II compartments. J Cell Biol. 1995;131(2):351–69. 7593164 10.1083/jcb.131.2.351 PMC2199989

[89] Altier C. Genetic and environmental control of salmonella invasion. Journal of microbiology. 2005;43 Spec No:85–92. . 15765061

[90] Jones BD. Salmonella invasion gene regulation: a story of environmental awareness. Journal of microbiology. 2005;43 Spec No:110–7. . 15765064

[91] Gross O. Measuring the inflammasome. Methods Mol Biol. 2012;844:199–222. doi: 10.1007/978-1-61779-527-5_15 . 22262445 10.1007/978-1-61779-527-5_15

