# Supplementary figures and images for: Increased autophagic sequestration in adaptor protein-3 deficient dendritic cells limits inflammasome activity and impairs antibacterial immunity
Source: PLoS Pathog. 2017 Dec 18;13(12):e1006785. doi: 10.1371/journal.ppat.1006785 (PMC5749898; doi:10.1371/journal.ppat.1006785)

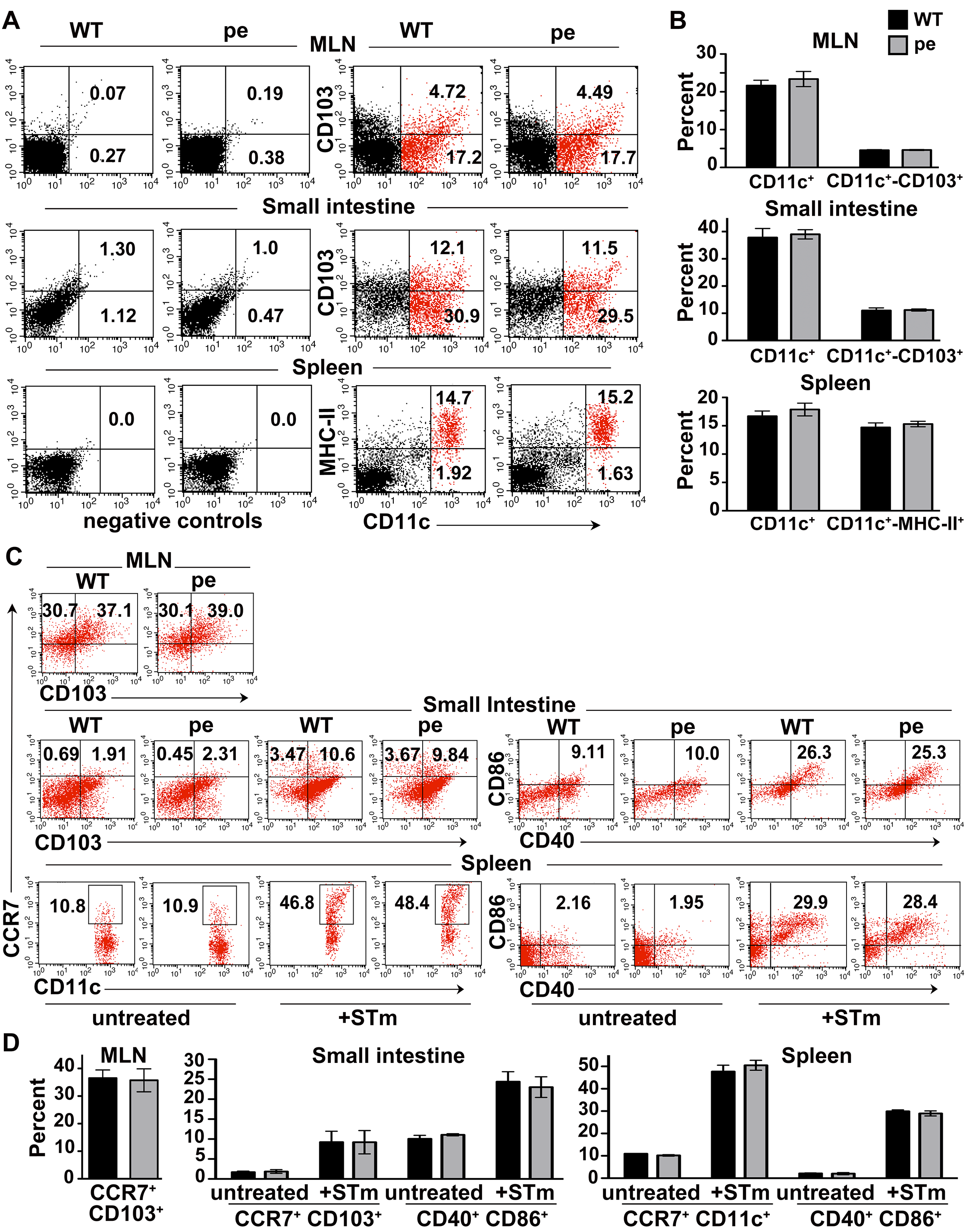

Supplement: S1 Fig — Phenotypic characterization of MLN, small intestine lamina propria and splenic DCs isolated from WT and AP-3-/- (pe) mice. (A, B) Cell suspensions from MLN (upper panels), small intestine lamina propria (middle panels), and B and T cell-depleted spleen (lower panels) were analyzed by flow cytometry. A. Representative dot-plots showing percentage of CD11c+ (red dots) and CD103+ populations in MLN and lamina propria, and CD11chi and MHC-II+ populations in spleen (right), compared to unstained negative controls (left). B. Data from three independent experiments presented as mean ± SD. No significant differences were detected. Average total cell numbers: 10x106 (MLN), 30x106 (small intestine), and 150x106 (spleen; 5x10^6^ after depletion). (C, D). Gated CD11c+ cells from MLN (upper panels), small intestine (middle panels) and spleen (bottom panels) from WT and pe mice. Cells were left untreated or infected with non-flagellin expressing STm for 6 h to induce cell maturation and prevent cell death. C. Representative dot-plots showing percentage of CCR7+ CD103+ (top left panels) and CCR7+ CD11c+ (bottom left panels), or CD86+ CD40+ (right panels) cells. D. Data from three independent experiments presented as mean ± SD. No significant differences were detected between WT and AP-3-/- cells. (TIF) [file ppat.1006785.s001.tif]

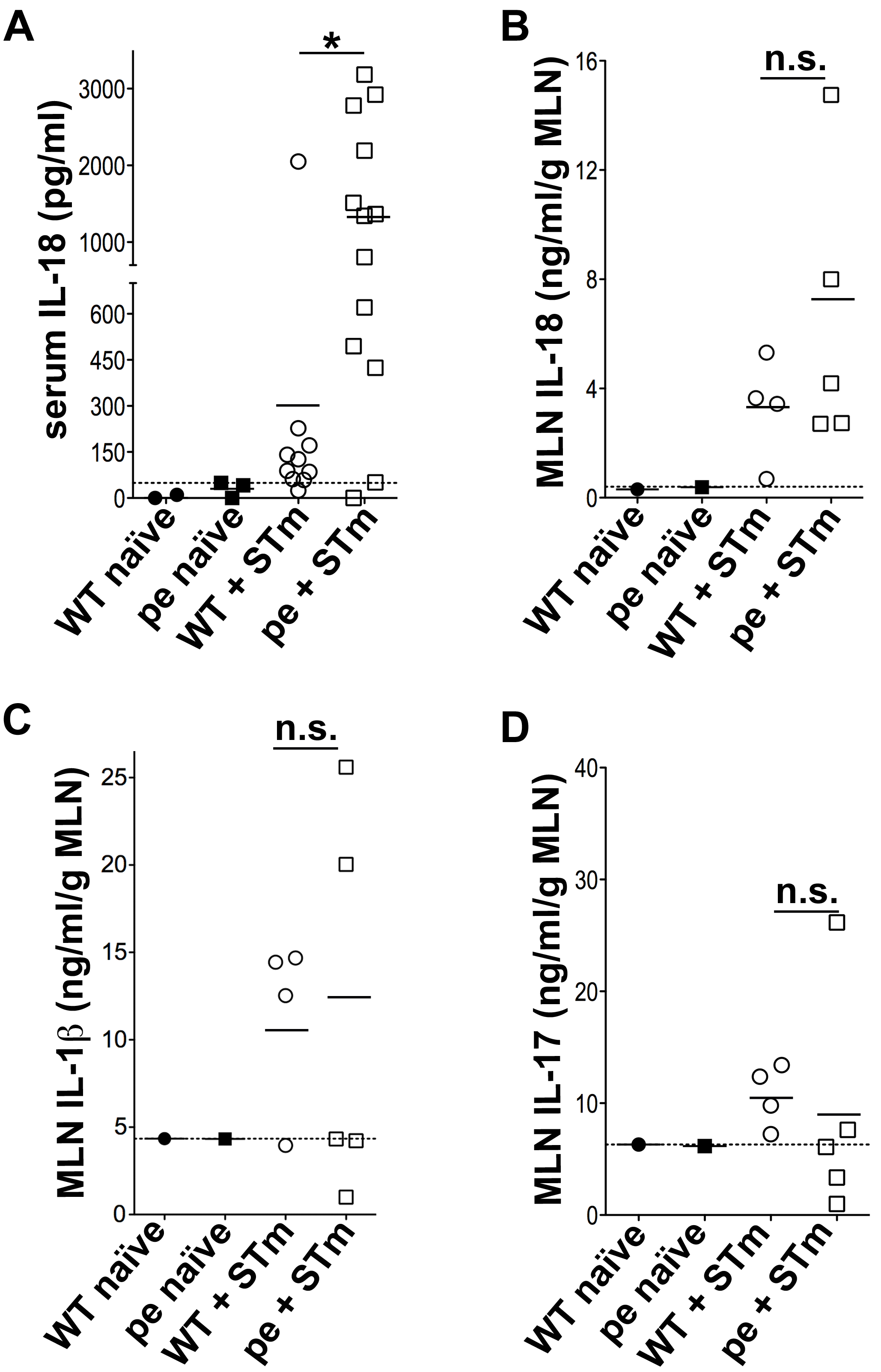

Supplement: S2 Fig — WT and pearl (pe) mice were infected orally with 108 STm (+ STm) or treated with PBS as a control (naïve), and analyzed five days after infection. A. Blood was collected by cardiac puncture, and serum was isolated and assayed for IL-18 by ELISA. Data are pooled from three independent experiments and expressed as pg IL-18/ ml serum. B-D. Supernatants from homogenized and pelleted MLN were assayed for IL-18 (B), IL-1β (C) and IL-17 (D) in one experiment. Dotted lines, background signal threshold from uninfected mice; solid lines, mean value. *p<0.05; n.s., not significant. (TIF) [file ppat.1006785.s002.tif]

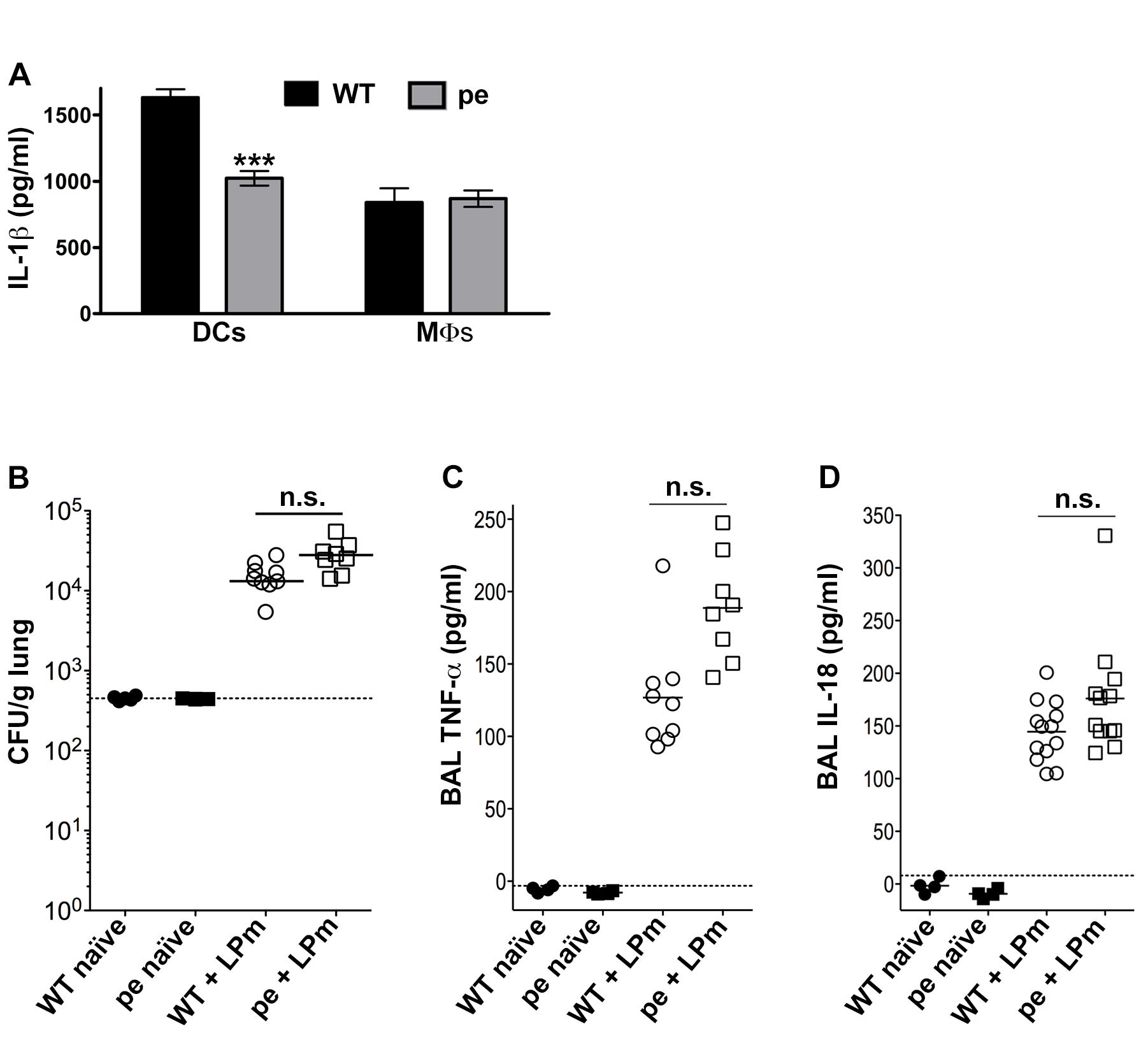

Supplement: S3 Fig — A. BMDCs (DCs) or BMMΦs (MΦs) from WT and pearl (pe) mice were infected with STm at a MOI of 10:1. Cell supernatants collected after 4 h were assayed for IL-1β by ELISA. (B-D) WT and pearl (pe) mice were infected intranasally with 5 × 10^6^ L. pneumophila ΔflaA (+ LPm) or received PBS as control (naïve). B. Lung homogenates were plated to measure bacterial load, expressed as CFU/ g of lung. (C, D). Bronchoalveolar lavage (BAL) was assayed for TNFα (D) or IL-18 (E) by ELISA. (B-D). Dotted lines, background (threshold values from uninfected mice); solid lines, geometric mean (B), or arithmetic mean (C, D) of values above background. ***p<0.001; n.s., not significant. (TIF) [file ppat.1006785.s003.tif]

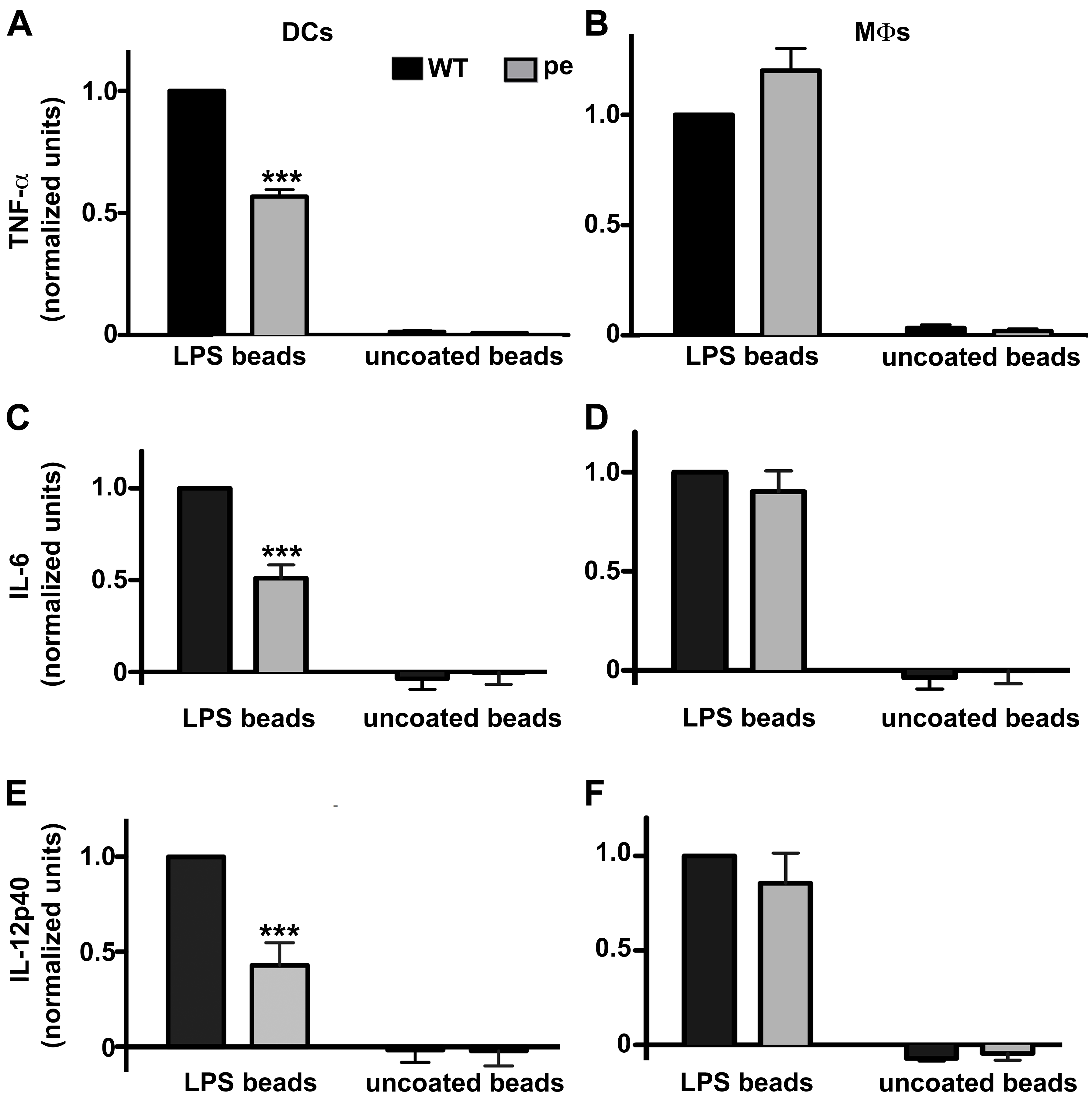

Supplement: S4 Fig — BMDCs (A, C, E) or BMMΦs (B, D, F) were incubated for 3 h with LPS-coated or uncoated latex beads, and TNFα (A, B), IL-6 (C, D) and IL-12p40 (E, F) were measured in cell supernatants by ELISA. Data from three independent experiments are normalized to LPS-coated bead-treated WT cells as 100% and represented as mean ± SD. ***p<0.001. (TIF) [file ppat.1006785.s004.tif]

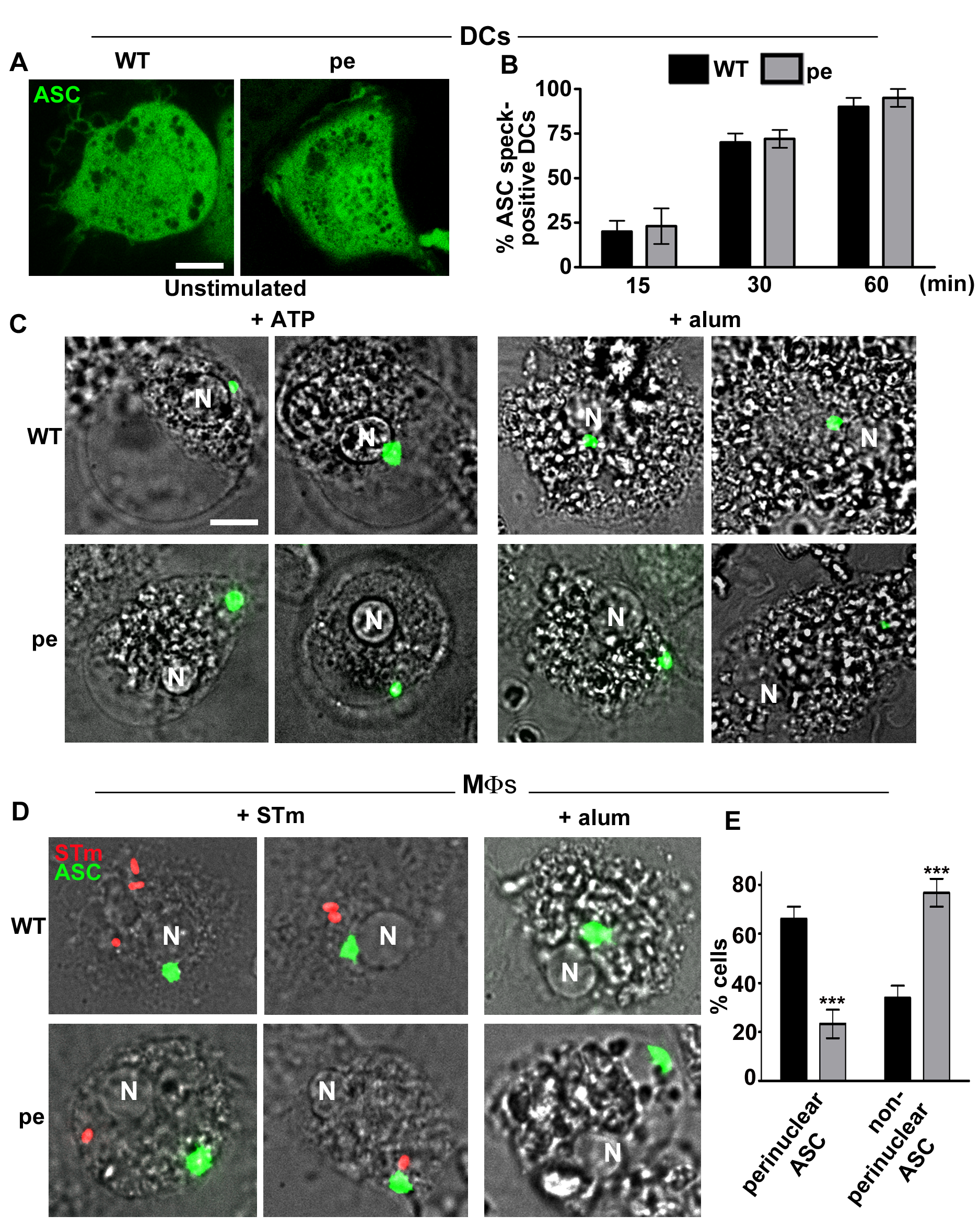

Supplement: S5 Fig — WT and pearl (pe) BMDCs (A-C) or BMMΦs (D, E) expressing ASC-GFP were analyzed by fluorescence microscopy. A. Representative images of uninfected BMDCs. B. BMDCs were infected with mCherry-STm and cells were analyzed at the indicated times after infection. ASC specks were quantified in 20 cells per cell type in each of three independent experiments. Data are presented as mean ± SD. No significant differences between WT and pearl cells were observed. C-E. BMDCs (C) or BMMΦs (D, E) were primed with LPS for 3 h and stimulated with ATP for 30 min (C left) or alum for 5 h (C, D right), or infected with STm for 1 h (D left). (C-D) Representative images showing ASC speck location (green) relative to whole cells visualized by DIC in WT and pearl cells. (E) Quantification of perinuclear (within a radius of one μμ from the nucleus) and non-perinuclear ASC specks in 15 BMMΦs per cell type in each of three independent experiments. N, nucleus. Scale bar: 10 μm. ***, p<0.001. (TIF) [file ppat.1006785.s005.tif]

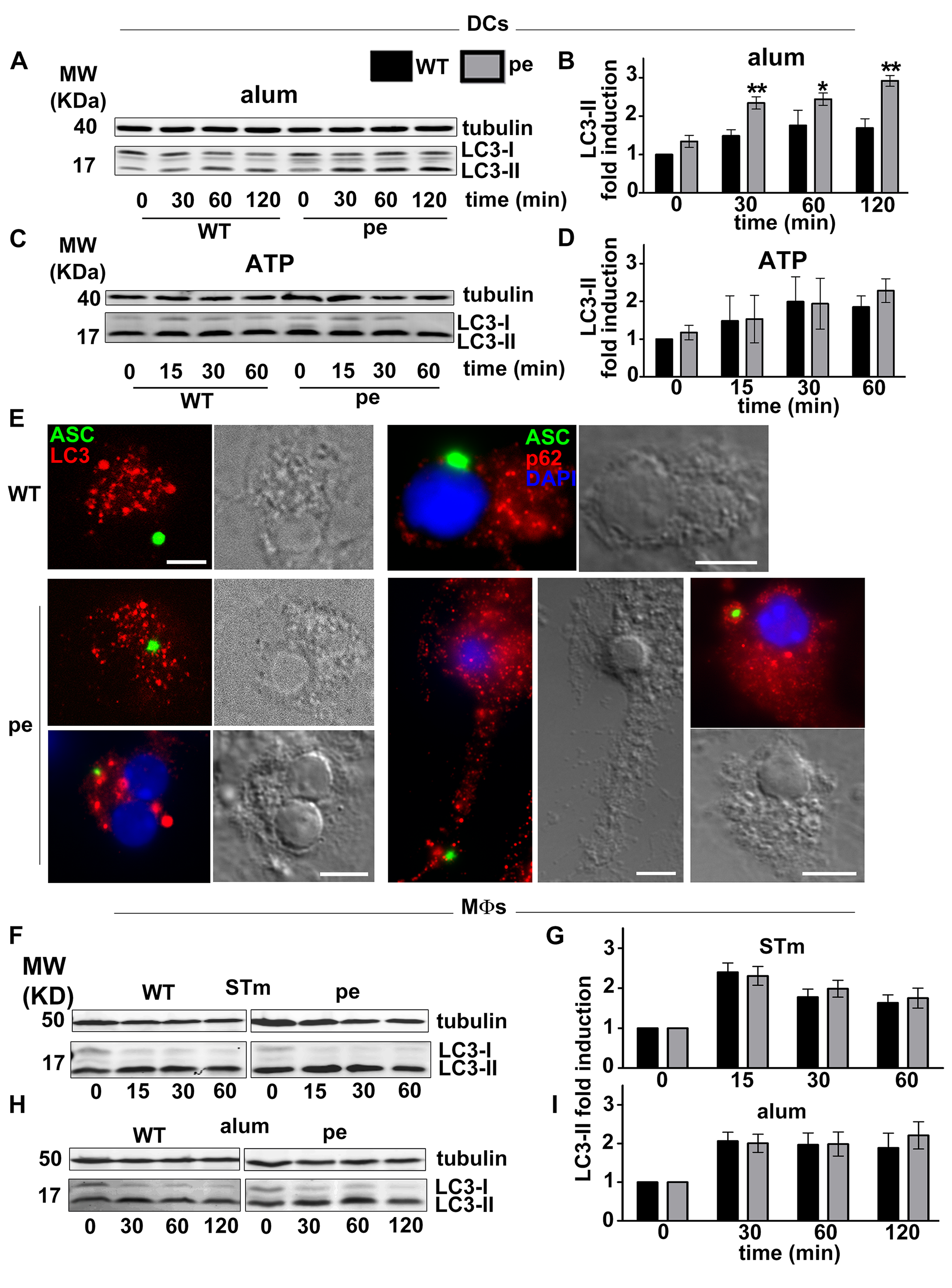

Supplement: S6 Fig — A-D, F-I. WT and pearl (pe) BMDCs (A-D) or BMMΦs (F-I) were primed with LPS for 3 h and stimulated with alum (A, B, H, I) or ATP (C, D), or infected with STm (F, G). Endogenous LC3 and β-actin or γ-tubulin as a loading control were detected by immunoblotting cell lysates at the indicated time points. A, C, F, H. Shown are representative blots, with the actin, tubulin and LC3-I and LC3-II bands highlighted. Positions of nearby molecular weight markers (MW) are shown to the left. B, D, G, I. Quantification of LC3-II band intensities from three independent experiments, expressed as fold increase relative to unstimulated cells and normalized to LC3-I and either β-actin or γ-tubulin levels. E. WT and pearl (pe) BMDCs expressing ASC-GFP alone or with mCherry-LC3 were infected with STm and analyzed by live imaging (for LC3) or immunofluorescence microscopy on fixed cells (for p62) 1 h later. Representative images showing ASC speck (green) and either LC3 puncta (red, left panels) or endogenous p62 puncta (red) relative to the nucleus labeled with DAPI (right panels) in infected cells. DIC images are shown to emphasize nuclear position. Data represent mean ± SD. Scale bars: 10 μm. *p<0.05; **p<0.01. (TIF) [file ppat.1006785.s006.tif]

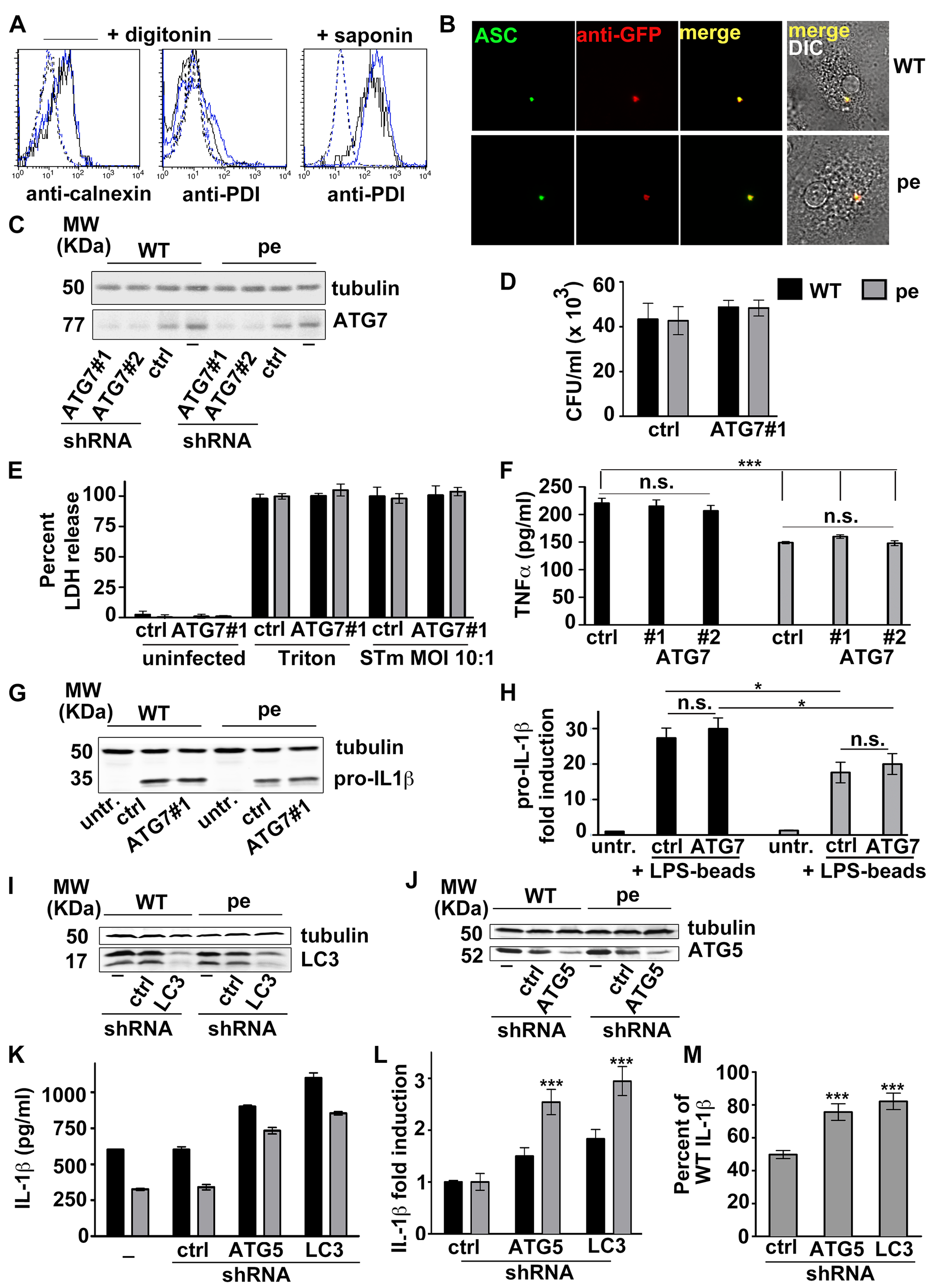

Supplement: S7 Fig — (A, B). WT and pearl (pe) BMDCs expressing ASC-GFP were infected with flagellin-expressing STm (to stimulate NLRC4 inflammasome) for 1 h, then treated either for 1 min with 50 μg/ml digitonin or throughout labeling with 0.1% saponin. A. Cells were then stained with a rabbit antibody to the cytoplasmic tail of the ER transmembrane protein, calnexin, and APC-conjugated anti rabbit antibody, or with a mouse antibody to the ER luminal enzyme, protein disulfide isomerase (PDI), and APC-conjugated anti mouse antibody. Cells were analyzed by flow cytometry; representative histogram plots are shown. Black lines, WT; blue lines, pe. Dotted lines, secondary antibody alone. B. WT (top) or pearl (pe, bottom) BMDCs expressing ASC-GFP were fixed and stained with AF594-labeled anti-GFP antibody and analyzed by IFM. Shown are individual and merged fluorescence images and corresponding DIC image at right. (C-J) WT and pearl (pe) BMDCs that were non-transduced (-) or transduced with lentiviruses encoding non-target (ctrl) or either of two ATG7-specific or ATG5- or LC3b- specific shRNAs were untreated (C, I, J) or infected with flagellin-expressing STm (MOI 10:1) (D-F), stimulated with LPS-beads (G, H), or primed for 3 h with LPS and stimulated with alum (K-M). (C, I, J). Representative immunoblots of cell lysates for ATG7, ATG5, LC3b or tubulin as a loading control. D. After 2h, cells were lysed with 0.5% Triton X-100 in PBS and serial dilutions were plated in streptomycin containing agar plates to assess colony formation. Data from 3 independent experiments are shown. E. After 2 h, cells were pelleted and LDH release into the supernatant was measured. Percent of LDH release was normalized to release from uninfected cells by 1% Triton X-100 treatment (Triton), and LDH release from uninfected cells was subtracted as background. Data from 3 independent experiments are shown F. Cell supernatants collected 2 h after Stm infection were assayed for TNFα by ELISA. (G, H) Cells were stimulat [file ppat.1006785.s007.tif]
